# Peripheral and central inflammation associated with progressive cognitive decline in dementia with Lewy bodies

**DOI:** 10.1093/braincomms/fcag274

**Published:** 2026-07-14

**Authors:** Peter Swann, Maura Malpetti, Leonidas Chouliaras, Simon R White, Elijah Mak, Ajenthan Surendranathan, P Simon Jones, Li Su, George Savulich, Stacey Kigar, Anna McKeever, Tim Fryer, Young T Hong, Franklin I Aigbirhio, James B Rowe, John T O’Brien

**Affiliations:** Department of Psychiatry, University of Cambridge, Cambridge CB2 0QQ, UK; Department of Clinical Neurosciences and Cambridge University Hospitals NHS Trust, University of Cambridge, Cambridge CB2 0SZ, UK; UK Dementia Research Institute at University of Cambridge, Cambridge CB2 0XY, UK; Department of Psychiatry, University of Cambridge, Cambridge CB2 0QQ, UK; Department of Psychiatry, University of Cambridge, Cambridge CB2 0QQ, UK; Department of Psychiatry, University of Cambridge, Cambridge CB2 0QQ, UK; Department of Radiology, Mayo Clinic, Rochester, MN 55905, USA; National Hospital for Neurology and Neurosurgery, University College Hospitals London, London WC1N 3BG, UK; Department of Clinical Neurosciences and Cambridge University Hospitals NHS Trust, University of Cambridge, Cambridge CB2 0SZ, UK; Department of Psychiatry, University of Cambridge, Cambridge CB2 0QQ, UK; Division of Neuroscience, Faculty of Health, School of Medicine and Population Health, University of Sheffield, Sheffield S10 2HQ, UK; Department of Psychiatry, University of Cambridge, Cambridge CB2 0QQ, UK; Department of Psychiatry, University of Cambridge, Cambridge CB2 0QQ, UK; Department of Medicine, University of Cambridge, Cambridge CB2 0AW, UK; Department of Psychiatry, University of Cambridge, Cambridge CB2 0QQ, UK; Department of Clinical Neurosciences and Cambridge University Hospitals NHS Trust, University of Cambridge, Cambridge CB2 0SZ, UK; Wolfson Brain Imaging Centre, Department of Clinical Neurosciences, Cambridge CB2 0QQ, UK; Department of Clinical Neurosciences and Cambridge University Hospitals NHS Trust, University of Cambridge, Cambridge CB2 0SZ, UK; Wolfson Brain Imaging Centre, Department of Clinical Neurosciences, Cambridge CB2 0QQ, UK; Department of Clinical Neurosciences and Cambridge University Hospitals NHS Trust, University of Cambridge, Cambridge CB2 0SZ, UK; Wolfson Brain Imaging Centre, Department of Clinical Neurosciences, Cambridge CB2 0QQ, UK; Department of Clinical Neurosciences and Cambridge University Hospitals NHS Trust, University of Cambridge, Cambridge CB2 0SZ, UK; Medical Research Council Cognition and Brain Sciences Unit, Cambridge CB2 7EF, UK; Department of Psychiatry, University of Cambridge, Cambridge CB2 0QQ, UK

**Keywords:** dementia, inflammation, Lewy, cognition, longitudinal

## Abstract

Dementia with Lewy bodies (DLB) is the second most common cause of neurodegenerative dementia, pathologically defined by the presence of Lewy bodies. Peripheral and central inflammation are increasingly recognized in DLB in clinical, post-mortem and animal studies. Finding clinically relevant biomarkers of inflammation in DLB will support the identification of novel pathways for disease-modifying therapies or use in clinical trials of immunomodulatory agents. Whilst there are cross-sectional studies of inflammation markers in DLB, there is limited evidence on the association between these markers and cognitive decline over time. Twenty participants with DLB underwent blood sampling for serum inflammatory markers, paired with PET imaging of the translocator protein (TSPO) and up to 4 years of longitudinal cognitive testing. Thirty participants with Alzheimer’s disease—comprising both Alzheimer’s dementia and/or mild cognitive impairment with biomarker evidence of amyloid pathology (AD/MCI+)—and 28 controls were also recruited for group comparisons. Data from 42 baseline cytokine immunoassays and TSPO PET were used as predictors of longitudinal cognitive scores in linear mixed-effects models. Partial least squares regression was used to test the association between peripheral and central inflammation. Using peripheral inflammatory markers as single predictors, we identified 14 associated with either a slower or faster rate of cognitive decline in DLB, whilst no single marker was predictive of decline in AD/MCI+. As many inflammatory markers were highly correlated, we used principal component analysis to identify a cytokine component associated with reduced cognitive decline in both DLB and AD/MCI+, that overlapped with the single markers identified in the previous analysis. A separate component was associated with cognitive decline in AD/MCI+ or DLB with Alzheimer’s dementia co-pathology (ascertained by amyloid PET). Widespread TSPO binding was associated with reduced cognitive decline in DLB, whilst a fronto-temporal pattern was associated with more rapid cognitive decline in both DLB and AD/MCI+. There were associations between peripheral cytokines and TSPO PET in AD/MCI+, but these were not significant in DLB. Overall, peripheral and central inflammation predicted cognitive decline in DLB. Specific patterns associated with both faster and slower rates of decline were identified. These profiles had both overlapping and contrasting associations when compared to AD/MCI+. Collectively, these data add to a body of evidence suggesting clinically relevant levels of inflammation in DLB. Future studies in larger, multi-site cohorts with multiple biomarker sampling points are required to understand the impact and dynamics of inflammation across all stages of the disease.

## Teaser

Swann *et al*. report patterns of peripheral and neuroinflammation associated with different rates of cognitive decline in dementia with Lewy bodies. The authors conclude that inflammation may be clinically relevant in dementia with Lewy bodies and explored as a future treatment target.

## Graphical Abstract

**Graphical Abstract:**
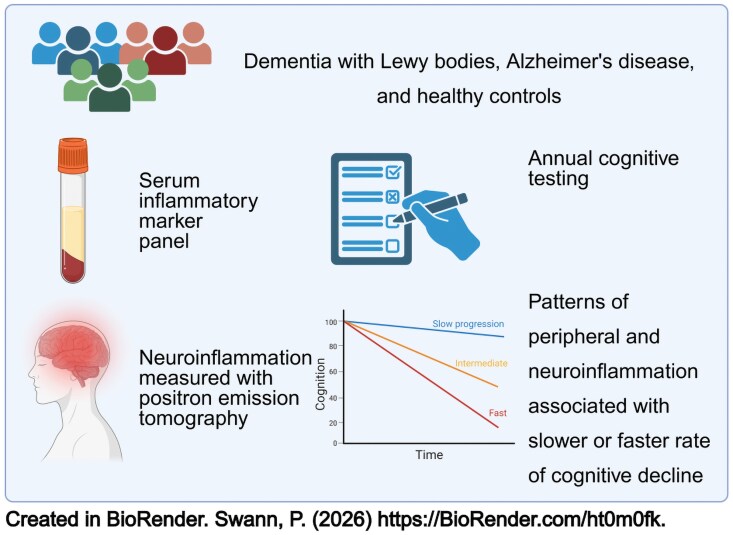

## Introduction

Dementia with Lewy bodies (DLB), the second most common cause of neurodegenerative dementia in older people,^1-3^ is diagnosed by the presence of core clinical features including visual hallucinations, REM sleep behaviour disorder, cognitive fluctuations, parkinsonism and indicative biomarkers.^4^ The pathological hallmarks are Lewy bodies, comprised of α-synuclein^5^ although there is frequent Alzheimer’s co-pathology of amyloid plaques and to a lesser extent neurofibrillary tangles.^6^ DLB is associated with increased mortality^7^ and impaired quality of life^8^ compared to Alzheimer’s disease. There are currently no disease-modifying treatments though potential targets include alpha-synuclein aggregation, amyloid, tau pathology and inflammation.^9,10^

Altered peripheral and central immune responses are now well recognized in neurodegenerative disorders. This is well established in Alzheimer’s disease^11^ with several identified risk genes enriched in microglia and immune pathways^12^ and a specific microglial response to Alzheimer's disease pathology detectable both *in vivo* using brain imaging^13,14^ and at post-mortem.^15^ Both innate and adaptive immune system changes are increasingly recognized in DLB.^16^ An immune response is induced by α-synuclein both *in vitro* and in animal models.^17^ There is evidence of cortical recruitment of T-lymphocytes in post-mortem tissue, with microglial activation only in the context of significant Alzheimer's disease co-pathology.^18^ There are completed and ongoing trials of anti-inflammatory and immune modulatory drugs in Parkinson’s disease, including azathioprine, montelukast and novel inflammasome inhibitors,^19^ and in Alzheimer's disease, including low-dose IL-2 therapy and immune checkpoint inhibitors.^20^ Vaccination against the neurotropic virus herpes zoster has been associated with a significant reduction in the risk of developing dementia. Although immunomodulatory drugs are well tolerated in Alzheimer's disease,^21^ so far this has not translated into favourable outcomes in clinical trials.^22,23^ This underscores the need for biomarker development both to enable stratification of patient groups most likely to respond to immunomodulatory drugs and for use in clinical studies to determine target engagement and monitor therapeutic response.

Cytokines, the major signalling proteins of the immune system, are increasingly of interest as biomarkers for patient stratification or targets in autoimmune diseases, infection and malignancy.^24^ Increased levels of serum cytokines have been identified at the prodromal and early stages of DLB^25,26^ with higher levels of IL-6 and TNF-α associated with greater cognitive and motor impairment. Peripheral cytokine profiles have been shown to predict disease progression in Parkinson’s disease^27^ and cognitive decline in Alzheimer's disease.^28,29^ One study found that a reduction over time in six cytokines (IFN-γ, IL-1β, IL-2, IL-4, IL-6 and IL-10) and their rate of change correlated with decreased cognitive scores in mild cognitive impairment (MCI) including MCI-LB (MCI with Lewy bodies).^30^

Other proposed novel therapeutics in neurodegeneration target microglial activation.^19,31^ PET brain imaging with tracers, such as [^11^C]-PK11195, that bind the translocator protein (TSPO) expressed in microglia,^32^ allows *in vivo* measurement of central inflammation. There is evidence of increased regional TSPO binding in early DLB^25^ and in Alzheimer's disease which correlates with pathology^13,14^ and cognitive decline.^33,34^ In Parkinson's disease, TSPO binding is associated with dementia risk,^35^ but its role in the progression of DLB has not been explored. Clinical^36^ and neuropathology studies suggest the immune response in DLB may vary depending on the presence of Alzheimer's disease co-pathology, particularly microglial activation.^37^

Neuroimaging of Inflammation in Memory and Related Other Disorders (NIMROD) is a deep phenotyping study measuring baseline peripheral inflammation with serum cytokine profiles and neuroinflammation with [^11^C]-PK11195 TSPO PET.^38^ Previous work from the NIMROD cohort has shown baseline differences in cytokine profiles and TSPO expression in DLB^25^ and that baseline TSPO binding predicts longitudinal cognitive decline in Alzheimer's disease.^33^

In this study, we included the longitudinal follow-up data with baseline inflammatory profiles to identify whether a specific peripheral or central inflammatory process predicts cognitive decline in DLB and whether the inflammatory profiles vary with the presence of Alzheimer’s co-pathology. We compared this to inflammatory profiles and cognitive decline in early clinical Alzheimer’s disease and tested for associations between central and peripheral cytokines. We hypothesized that specific immune profiles would be associated with cognitive decline in DLB compared to Alzheimer's disease and that in DLB differences in profiles would be seen depending on the presence or absence of Alzheimer's disease co-pathology.

## Materials and methods

### Participants

Participants were recruited to the NIMROD cohort as previously described^25,38^ and gave informed consent in line with the Declaration of Helsinki. The NIMROD study was approved by the Cambridge Central Research Ethics Committee. Participants with a clinical diagnosis of probable DLB as defined by consensus criteria^4^ were recruited for TSPO PET imaging and serum inflammatory marker measurements (*n* = 20). In addition, participants with either MCI due to Alzheimer’s disease^39^ as indexed by a positive amyloid PET scan (cut-off 19 centiloids,^40^  *n* = 17) or a clinical diagnosis of Alzheimer’s dementia according to standard criteria^41^ (*n* = 16) were recruited and subsequently referred to as the AD/MCI+ group (*n* = 30 with TSPO PET, *n* = 26 with inflammatory marker measurements, and *n* = 25 with both). Control participants with the absence of cognitive symptoms and Mini-Mental State Examination > 26/30 also underwent baseline serum inflammatory marker analysis (*n* = 28 with inflammatory marker measurements, *n* = 16 with both TSPO PET and inflammatory marker measurements). Control participants were recruited based on the absence of cognitive symptoms or evidence of cognitive impairment on neuropsychological testing^38^ and followed up with repeat cognitive and clinical assessments without showing any evidence or reporting symptoms of cognitive decline (93% with a minimum 1-year follow-up, median follow-up = 2.95 years, IQR = 1.17–3.24 years). The study excluded those with acute infection, systemic inflammatory disorders, those on immunosuppressants, or major psychiatric or any other neurologic conditions. Participants underwent neuropsychological testing and symptom rating scales including the Movement Disorders Society Unified Parkinson’s Disease Rating Scale (MDS-UPDRS) Part III,^42^ Neuropsychiatric Inventory (NPI) ^43^ and One Day Fluctuation Scale (ODFS).^44^

### Cognitive assessment

Cognition was measured with the Addenbrooke’s Cognitive Examination Revised (ACE-R),^45^ a cognitive examination with utility in both DLB and Alzheimer's disease.^46^ DLB participants were followed up for repeat cognitive testing at approximately 1- year intervals up to 4 years (median length of follow-up 2 years, interquartile range 1.6–2.7 years). All blood samples were taken within 6 months of baseline testing (median = 2 months). AD/MCI+ participants were followed up for cognitive testing at 1- year intervals up to 5 years (median length of follow-up 3 years, interquartile range 2–3.5 years). We defined as baseline the visit at which the participants underwent blood sampling. Blood samples were taken within 6 months of baseline testing (except for 1 at 7 months, median = 2 months). The NIMROD study had yearly follow-up cognitive testing up to 4 years. Participants also consented to the sharing of clinical cognitive test scores to maximize the follow-up data available. As anticipated, the proportion of dropouts increased with study duration (DLB at 1 year: 5%, 2 year: 30%, 3 years: 60%, AD/MCI+ at 1 year: 9%, 2 year: 27%, 3 years: 36%). The reasons for dropping out in DLB were as follows: 40% completed 3 year follow-up, 25% deceased, 25% untestable, 5% declined follow-up and 5% lost to follow-up. In AD/MCI+, 63% completed 3 years follow-up, 25% declined further follow-up, 6% untestable, 3% deceased and 3% lost to follow-up.

### Blood sampling and analyses

Blood was collected at baseline by venepuncture in serum gel tubes. After 30 min at room temperature to allow clotting, samples were centrifuged. Serum was aliquoted and stored at −70° prior to analysis. Inflammatory marker assays were performed at the Core Biochemical Assay Laboratory at Cambridge University Hospitals NHS Foundation Trust. Forty-one cytokines, chemokines and related inflammatory markers were measured (Mesoscale Discovery V-Plex Human Cytokine panel and separate assays for hsCRP, IL-34, YKL-40 and MCSF1), as detailed in Supplementary Table 1. Assays were performed in duplicate as per the manufacturer’s instructions and the mean result reported. For plate-based assays (all measurements excluding hsCRP), within each plate, three quality control standards were analysed at the beginning and end of each plate. These standards were comprised of either serum or plasma pools, or stock calibration solution and spanned the range of expected concentrations in the assay. The Siemens Dimension EXL autoanalyzer is calibrated daily with a commercial quality control standard for the measurement of hsCRP.

### PET imaging acquisition and processing

[^11^C]-PK11195 PET imaging was performed as previously described.^25^ In brief, this was performed on a GE Advance PET scanner (GE Healthcare) or GE Discovery 690 PET/CT. Attenuation correction was provided by the associated MR or CT scan. Dynamic imaging was recorded in 55 frames over 75 min following a 500 MBq bolus injection [^11^C]-PK11195. Non-displaceable binding potential was quantified with a simplified reference tissue model^33^ for regions in the Hammers atlas^47^ with partial volume correction. We excluded the cerebellum, corpus callosum, frontal horn, temporal horn and third ventricle as regions of no interest. [^11^C]-PK11195 PET scans for the DLB group were performed on the day of bloods except for three participants (one at 3 months, one at 7 months and one at 9months). PET scans for the AD/MCI+ group were performed on the day of blood draw except for four participants (one at 8 months, one at 10 months, one at 11 months). One outlier in the AD/MCI+ group had 34 months between cytokine measurement and PET imaging, and the PET data were excluded from the analysis of cognitive decline. A subsample of DLB participants (*n* = 17) underwent PIB PET imaging as described previously, using a 550 MBq bolus and 30 min of imaging performed after a 40 min post-injection.^25^ Centiloids (CL) were calculated using standard methodology^48^ with a cut-off of 19 defining PET amyloid positivity for MCI cases^49^ In DLB, we used the centiloid as a continuous measure of increasing amyloid co-pathology, and a cut-off of 56 for amyloid positivity, as previously established as appropriate for PiB in DLB.^50^

### Statistical analysis

#### Demographics

Differences in age and ACE-R were compared between groups using the Kruskal–Wallis test, and differences between sex with *χ*².^51^

#### Imputation of missing inflammatory markers

Several inflammatory marker measurements had values that were below the limit of detection. To minimize biasing the data by excluding specific markers that were present only in low concentrations, without introducing bias by imputing large percentages of data, we excluded those with more than 50% missing data. This has been shown to produce acceptable results for variables remaining in the analysis following multiple imputation, including in skewed cytokine measures.^52^ Multiple imputation performs well for handling left censored data, compared to single value substitution.^53^ As the inflammatory marker values were left skewed, we imputed the tail of the distribution below the limit of detection from the triangle function.^54^ There were 11 markers with greater than 50% values below the lower limit of detection which were excluded. Nine had missing values which were imputed (Supplementary Table 2).

#### Linear mixed-effects models

For estimating rate of cognitive decline, we used linear mixed-effects models with ACE-R as the response variable and time as the predictor, with individual as a random effect.^55^ To measure the association of an inflammatory marker with cognitive decline, we included these as predictors in interaction terms with time. To generate comparable effect size estimates, values were scaled by interquartile range (as most were not normally distributed) prior to taking the log. Following imputation, we tested for the association of individual markers with ACE-R scores over time in the DLB and AD/MCI+ groups separately. We modelled the interaction between time (in years from baseline ACE-R score) and each inflammatory marker controlling for age, sex and baseline ACE-R. We included the individual as a random effect for intercepts, but due to the study size, we did not include random slopes.

For the inflammatory markers with missing values, the model was run for each iteration of imputed values (100 iterations). The results were pooled using Rubin’s rules.^56^ The interaction between time and the inflammatory marker of interest represents the strength of the association between the baseline marker value and longitudinal cognitive decline. *P*-values for the interaction were corrected for multiple comparisons by false discovery rate (FDR). Significant results were tested with The Kyoto Encyclopaedia for Genes and Genomes (KEGG)^57^ over-representation analysis using clusterprofiler^58^ to identify common pathways associated with cognitive decline.

#### Principal component analysis

We conducted a principal component analysis (PCA) using prcomp with a varimax rotation. This was run separately for the peripheral inflammatory measurements and TSPO binding potentials (volume-weighted means of left and right regional binding potentials). The number of components was selected by the elbow rule and Horn’s parallel analysis, including only components with an adjusted eigenvalue greater than one.^59^ Individual scores for each component were used as predictor variables in linear mixed-effects models.

#### Partial least squares regression

We included the 40 bilateral regions of TSPO expression as predictors and 30 peripheral inflammatory marker measurements as response variables in a partial least squares regression^60^ (PLS-R) model using cross validation and the Wold criteria to select the number of components, separately in DLB and AD/MCI+ groups. Confidence intervals for the regression coefficients from the PLS-R between PK regions and peripheral inflammatory markers were generated using the jack-knife method with associated *P*-values tested against the null hypothesis that the regression coefficient was not different from zero.

## Results

### Demographics

There were no significant differences in age and sex between DLB and controls, but the DLB group was numerically older and had a larger proportion of males. The AD/MCI+ group was older than controls. The DLB group had lower ACE-R scores than the AD/MCI+ group, with both groups having lower scores than controls (Table 1), and DLB participants were more functionally impaired as measured by the Bristol Activities of Daily Living Scale (BADLS). The median PiB PET CL in DLB participants was 76.1 (Q1 = 24.4, Q3 = 123.0), and using a cut-off at 56 CL^50^ 9/17 (53%) were amyloid-positive.

**Table 1:** Demographics table

	Control (*N* = 28)^a^	DLB (*N* = 20)^a^	AD/MCI+ (*N* = 31)^a^	*P*-value (overall)^b^	*P*-value (post-hoc)^c^
Age	69 (64, 72)	75 (69, 78)	75 (67, 79)	0.012	Control versus DLB: *P* = 0.053 Control versus AD/MCI+: *P* = 0.030 DLB versus AD/MCI+: *P* = 0.85
Sex				0.10	
Female	14 (50%)	4 (20%)	13 (42%)		
Male	14 (50%)	16 (80%)	18 (58%)		
ACE-R	96 (92, 98)	67 (57, 78)	80 (71, 85)	<0.001	Control versus DLB: *P* < 0.001 Control versus AD/MCI+: *P* < 0.001 DLB versus AD/MCI+: *P* = 0.035
MDS-UPDRS-III	0 (0, 0, 4 missing)	27 (19, 37)	1 (0, 2)	<0.001	Control versus DLB: *P* < 0.001 Control versus AD/MCI+: *P* = 0.083 DLB versus AD/MCI+: *P* < 0.001
NPI	NA	10 (3, 22)	4 (2, 9)	0.049	
ODFS	NA	3 (1, 4.5)	0.5 (0,2)	0.033	
BADLS	NA	14.5 (7, 22)	4 (1, 6)	<0.001	

Table showing age, sex and baseline assessments for the three groups.

^a^Median (Q1, Q3).

^b^Differences in sex compared with Pearson’s *χ*², differences in age, Addenbrooke’s cognitive examination revised (ACE-R) and Movement disorders society unified Parkinson’s disease rating scale III (MDS-UPDRS-III) with Kruskal–Wallis rank sum test, neuropsychiatric inventory (NPI), one day fluctuation scale (ODFS) and Bristol Activities of Daily Living Scale (BADLS) with Wilcoxon rank sum test.

^c^ *Post hoc* Dunn test with Benjamin–Hochberg correction following a significant Kruskal-Wallis test.

### Peripheral inflammatory markers are associated with cognitive decline in DLB

Using a linear mixed-effects model without covariates, the estimate for the association of time on ACE-R was −6.6 points per year for DLB and −5.6 points for AD/MCI+ (Supplementary Fig. 1). Box plots for inflammatory marker values between groups are displayed in Supplementary Fig. 2.

In the DLB group, IL15, YKL-40, IL6, IL12p70 and Eotaxin3 were significantly associated with a greater annual rate of cognitive decline over time after correction for multiple comparisons. MDC, MCSF1, IL12, IL16, TNFR1, MIP3alpha, GMCSF, TNF-beta and IP10 were significantly associated with a slower rate of cognitive decline (Table 2).

**Table 2:** Significant peripheral cytokine predictors of annual rate of cognitive decline in DLB, with estimates from linear mixed-effects models

Parameter	Estimate	*P*-value	*P*-value (FDR)
YKL40	−6.10	<0.005	<0.005
MCSF1	15.26	<0.005	<0.005
TNFR1	24.28	<0.005	<0.005
IL12	8.54	<0.005	<0.005
IL15	−33.71	<0.005	<0.005
IL16	13.34	<0.005	<0.005
TNFB	4.87	0.01	0.03
IP10	4.43	0.02	0.04
MDC	30.79	<0.005	<0.005
IL6	−6.60	<0.005	0.01
MIP3a	5.96	<0.005	<0.005
GMCSF	9.18	<0.005	0.01
Eotaxin3	−6.82	0.02	0.04
IL12p70	−4.12	<0.005	0.01

**Figure 1:**
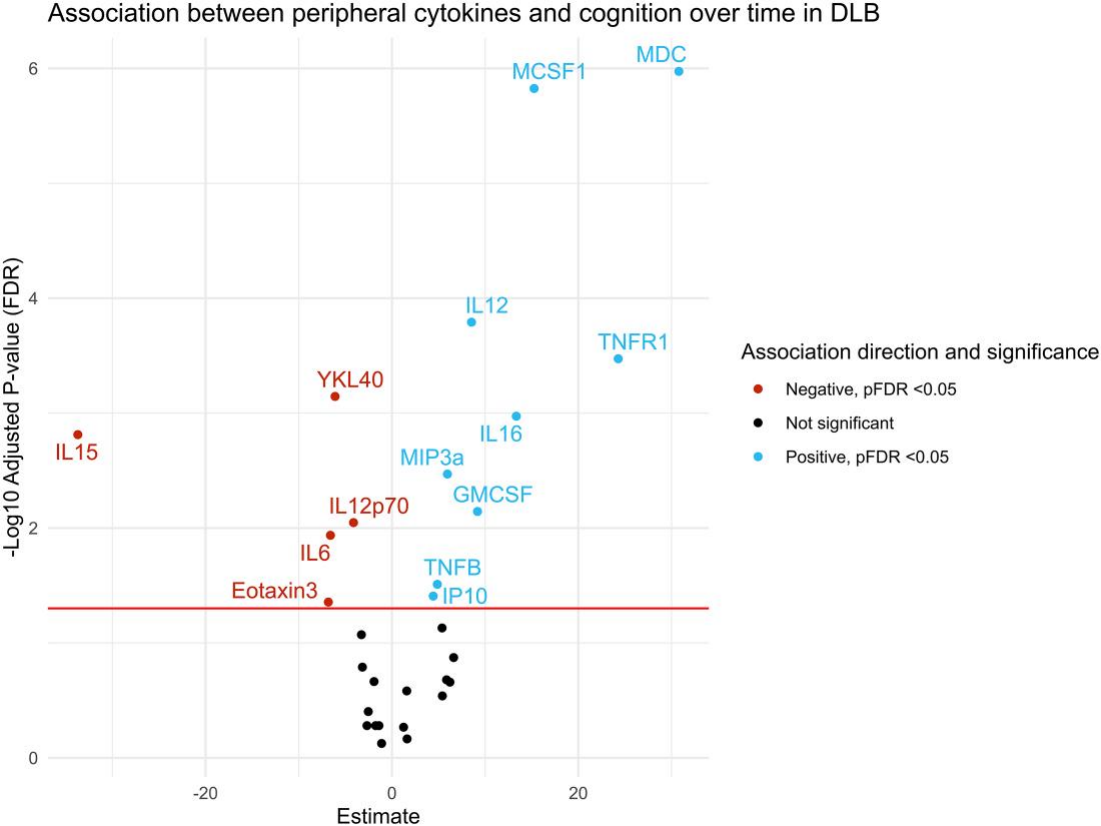
**Volcano plots showing the association of peripheral cytokines and cognitive decline in DLB.** Each data point represents the estimate (effect size) and *P*-value from the interaction between time and the peripheral inflammatory marker labelled as predictors of ACE-R, from separate linear mixed-effects models in DLB participants (*n* = 20). The linear mixed-effects models include age, sex and baseline ACE-R as covariates, with the individual as a random effect. The *y*-axis shows the FDR-corrected −log(*P*-value), and the red horizontal line shows the significance threshold of pFDR = 0.05. The *x*-axis shows the estimate (beta coefficient) from the linear mixed-effects model for each predictor, and the points are labelled with the cytokine name. The colours signify the strength and direction of association (red = associated with more rapid cognitive decline, significant at FDR *P* < 0.05, black = not significant, pale blue = associated with slower rate of cognitive decline, FDR *P* < 0.05). ACE-R, Addenbrookes Cognitive Examination Revised; DLB, dementia with Lewy bodies; FDR, false discovery rate; IL15, interleukin 15; IL6, interleukin 6; YKL40, chitinase-like-protein 3; IL12p70, interleukin 12; MIP3a, macrophage inflammatory protein-3-alpha; TNFB, tumour necrosis factor beta; IP10, inducible protein 10; GMCSF, granulocyte-macrophage colony stimulating factor; IL16, interleukin 16; IL12, p40 subunit of IL12/IL23; IL16, interleukin 16; MCSF1, macrophage colony stimulating factor; TNFR1, tumour necrosis factor receptor 1; MDC, macrophage derived chemokine.

In AD/MCI+, there were no peripheral inflammatory markers significantly associated with cognitive decline after correction for multiple comparisons (Fig. 1; Supplementary Tables 3 and 4). To identify common dysregulated pathways, significant results were tested for overrepresentation within known biological processes in the KEGG database. In DLB, significantly overrepresented pathways included cytokine–cytokine receptor interactions, viral protein interactions, TNF signalling pathway, IL-17 signalling pathway and the JAK-STAT signalling pathway (Supplementary Figs 3 and 4).

### PCA identifies three peripheral cytokine profiles

As many inflammatory markers were highly intercorrelated, we conducted a PCA including DLB, AD/MCI+ and controls. This was performed in order to reduce the data to components capturing the most variance, to test whether a pattern of peripheral inflammation was associated with cognitive decline.

**Figure 2:**
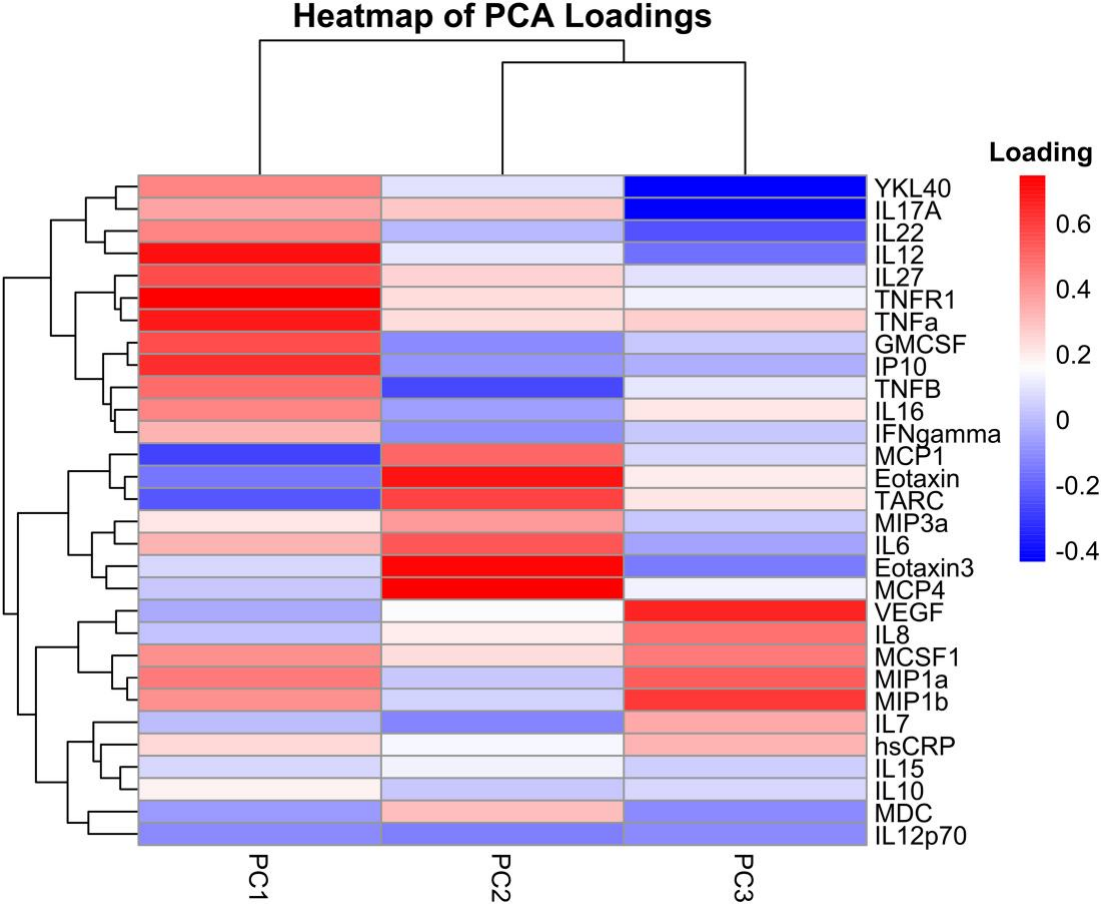
**Heatmap showing the loadings of the three components of cytokines.** Each column represents a principal component (PC1 = principal component 1, PC2 = principal component 2, PC3 = principal component 3) and each row a peripheral inflammatory marker from a PCA analysis including all participants (*n* = 74). The colour of the cell represents the loading (unitless) of each cytokine on that component, with red indicating strong positive loading and blue indicating strong negative loading. The dendrogram indicates the clustering between cytokines (rows) or components (columns) based on Euclidean distance.

The PCA identified three main components explaining 34% of the variance in the data. The major loadings for the first component (values greater than 0.5) were TNFR1 (0.74), IL-12 (0.70), TNF-alpha (0.67), IP10 (0.63), IL27 (0.57) and GMCSF (0.56). The major loadings for the second component were MCP4 (0.74), Eotaxin3 (0.72), Eotaxin-1 (0.69), TARC (0.59), IL-6 (0.53) and MCP-1 (0.50). The major loadings for the third component were VEGF (0.66), MIP1-beta (0.61) and MIP1-alpha (0.53). A full list of loadings is included in Fig. 2 and Supplementary Table 5. The loadings for components 1 and 3 were primarily negative, so they were multiplied by −1 to aid interpretability.

### Peripheral inflammatory profiles are associated with cognitive decline in DLB and AD/MCI+

**Figure 3:**
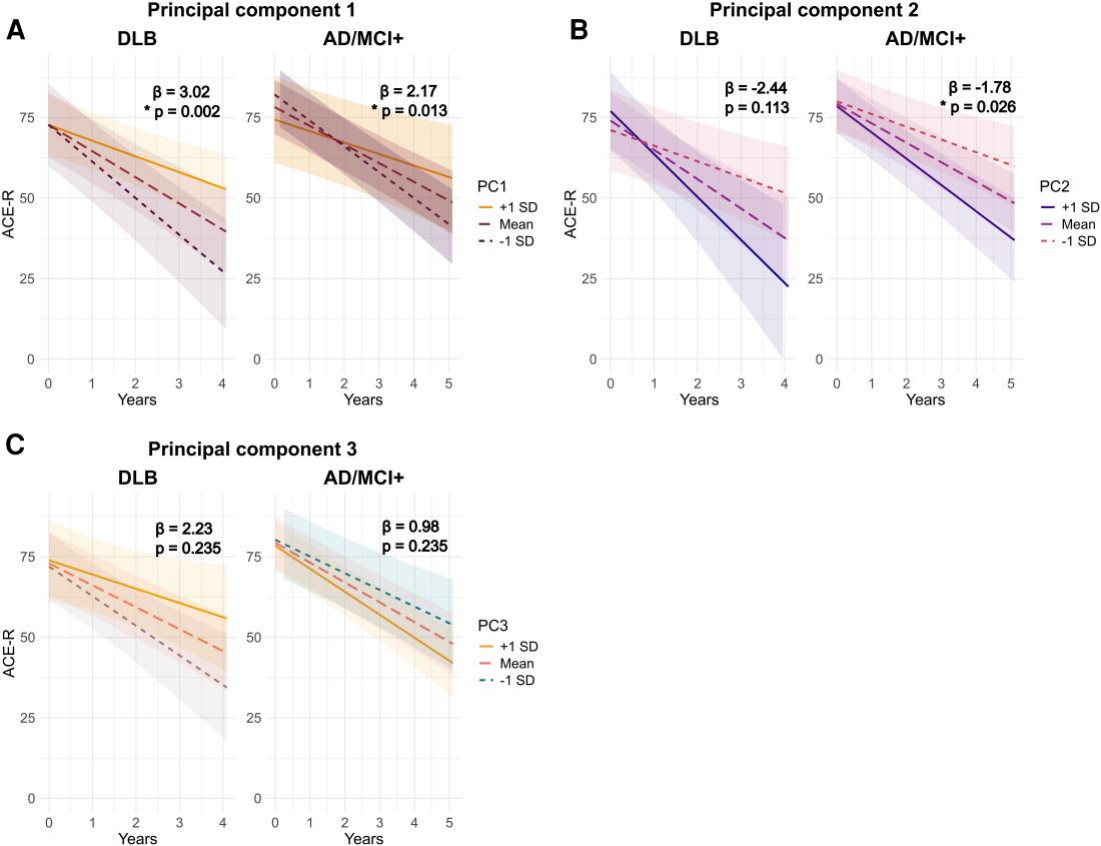
**Plots showing interaction between time and individual principal component scores of cytokines as predictors of ACE-R scores.** The *y*-axis shows ACE-R scores, and the *x*-axis shows time in years. The differences in the slope of the lines represent the difference in the rate of cognitive decline at representative varying inflammatory component scores, controlling for age, sex and baseline cognition in a linear mixed-effects models with the individual as the random effect (DLB *n* = 20, AD/MCI + *n* = 31). The lines shown represent the trajectories of the mean score, +1 and −1 SD, with 95% confidence intervals. The effect size, beta and *P*-value are shown for the interaction term. The plots are using exemplar data from the first imputed data set, with effect sizes and *P*-values pooled from 100 iterations. Increased PC1 scores are associated with slower rates of cognitive decline in both DLB and AD/MCI+ (**A**). Increased PC2 scores are associated with a greater rate of cognitive decline in AD/MCI+, but not DLB (**B**). There was no relationship between PC3 and cognitive decline in either group (**C**). ACE-R, Addenbrookes Cognitive Examination Revised; PC, principal component; DLB, dementia with Lewy bodies; AD/MCI+, Alzheimer’s dementia and mild cognitive impairment with evidence of amyloid pathology; SD, standard deviation.

Following the PCA, we extracted individual component scores and used these as predictor variables in linear mixed-effects models. To test for the association between these inflammatory components and cognitive decline, we used the interaction between individual component scores and time in a linear mixed-effects model, controlling for age, sex and baseline cognition, with the individual as a random effect (Fig. 3). In DLB, PC1 was a significant predictor of cognitive decline, with higher levels of PC1 associated with a slower rate of cognitive decline (interaction time * PC1, estimate 3.02, *P* = 0.002) whilst neither PC2 nor PC3 was significantly associated with the rate of cognitive decline (PC2: estimate = −2.44, *P* = 0.113, PC3: estimate = 2.23, *P* = 0.221). In AD/MCI+, higher levels of PC1 were also associated with a slower rate of cognitive decline (estimate 2.17, *P* = 0.013) whilst higher levels of PC2 were associated with a faster rate of cognitive decline (estimate −1.78, *P* = 0.026). There was no significant association with PC3 (estimate 0.98, *P* = 0.235) (Fig. 3; Supplementary Tables 6 and 7).

**Figure 4:**
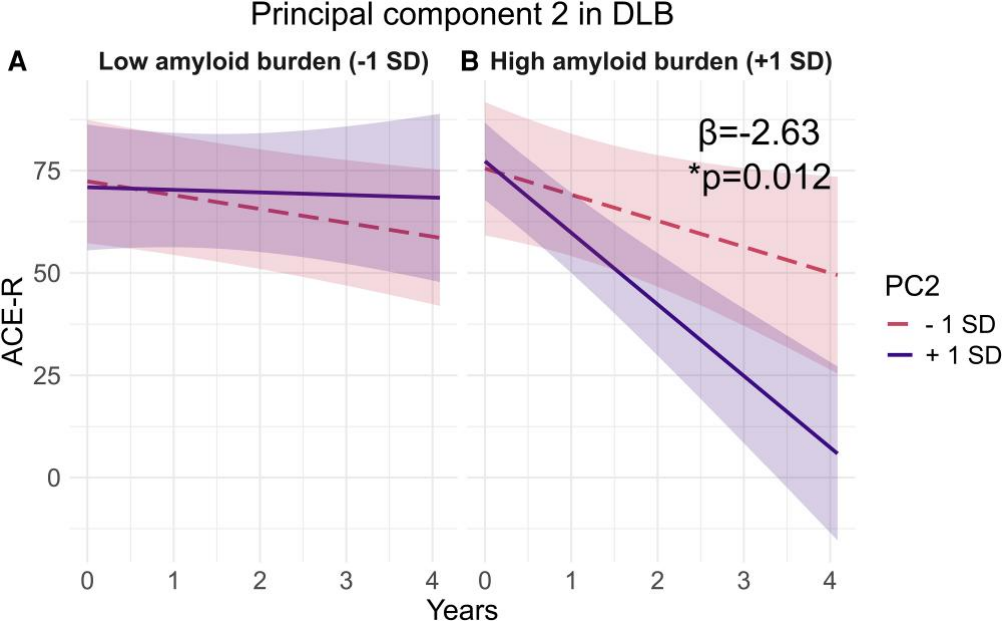
**Interaction plot showing that the relationship between principal component 2 scores and longitudinal cognition is dependent on amyloid co-pathology in DLB.** (**A**) In those with low amyloid burden, there are no differences in the rate of cognitive decline with either low (dashed red line) or high (solid purple line) levels of principal component 2 (PC2) scores. (**B**) In those with high amyloid burden, those with high levels of PC2 scores have a significantly greater rate of cognitive decline than those with low levels of PC2 (estimate −2.63, *P* = 0.012 for the interaction between time, PC2 and centiloid as predictors of ACE-R including age, sex and baseline ACE-R as covariates in a linear mixed-effects model, with individual as a random variable (*n* = 17). PC, principal component; DLB, dementia with Lewy bodies; SD, standard deviation. Lines are shown with 95% confidence intervals.

As PC2 was a significant predictor of more rapid decline in AD/MCI+, as an exploratory analysis, we included an additional interaction to test whether the association between PC2 and cognitive decline depended on the presence of amyloid co-pathology by adding centiloid values from PiB PET as an interaction term. There was a significant three-way interaction between time, PC2 and PiB status (estimate −2.63, *P* = 0.012) showing that with increasing centiloid, PC2 is significantly associated with a faster rate of cognitive decline. Using a binary split of amyloid-positive versus negative, however, this result was no longer significant (estimate = −1.88, *P* = 0.068) (Figure 4; Supplementary Table 8).

### Distinct patterns of [^11^C]-PK11195 binding predict cognitive decline in DLB and AD/MCI+

In order to examine whether a pattern of [^11^C]-PK11195 binding was associated with cognitive decline, we included partial volume corrected regional [^11^C]-PK11195 binding potentials in a PCA including DLB, AD/MCI+ and controls. The volume corrected means for bilateral lateral regions were used, and all groups were included in the PCA. We identified three main components explaining 63% of the data. We applied a varimax rotation to the loadings and extracted individual scores for each of the components.

**Figure 5:**
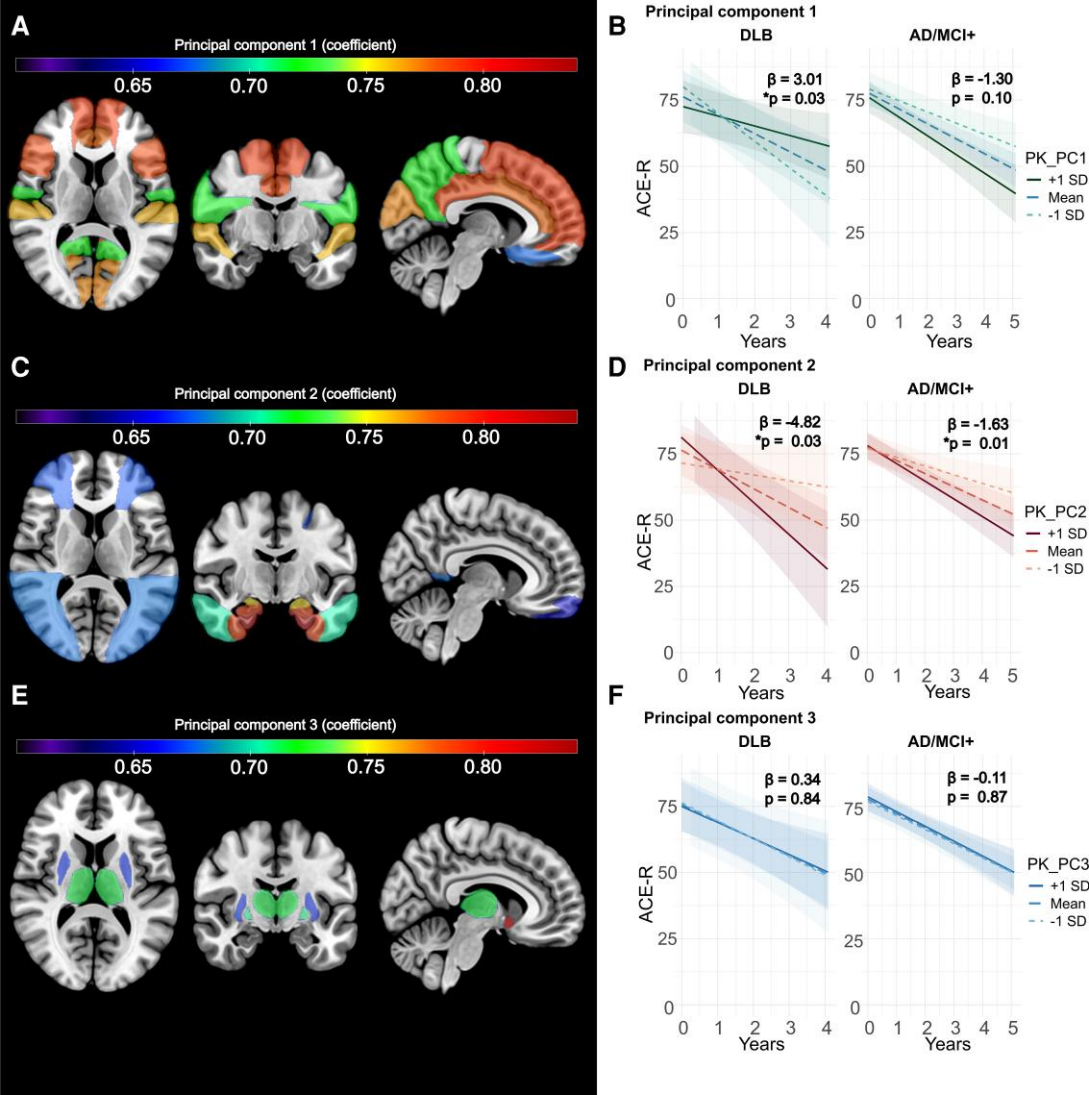
**Plots showing the [^11^C]-PK11195 principal component loadings across brain regions and the interaction between time and individual principal component scores of [^11^C]-PK11195 binding potentials as predictors of ACE-R scores.** [^11^C]-PK11195 principal component 1 (**A**), principal component 2 (**C**) and principal component 3 (**E**) loadings displayed as colour intensities on the MNI152 brain template. The colour represents the magnitude of the loading. Only regions with loadings greater than 0.6 are displayed. Interaction plots from linear mixed-effects models for the interaction between individual [^11^C]-PK11195 principal component 1 (**B**), principal component 2 (**D**) and principal component 3 (**F**) scores and time in predicting ACE-R score. The *y*-axis shows ACE-R scores, and the *x*-axis shows time in years. The differences in the slope of the lines represent the difference in the rate of cognitive decline at representative varying [^11^C]-PK11195 principal component 1 scores, controlling for age, sex and baseline cognition in a linear mixed-effects models with the individual as the random effect (DLB *n* = 20, AD/MCI+ *n* = 30). The lines shown represent the trajectories of the mean score, +1 and −1 standard deviation, with 95% confidence intervals. The effect size, beta and *P*-value are shown for the interaction term. Increased PC1 is significantly associated with a reduced rate of cognitive decline in DLB, but not AD/MCI+, Increased PC2 is significantly associated with an increased rate of cognitive decline in both DLB and AD/MCI+. There were no significant association with PC3. PC, principal component; DLB, dementia with Lewy bodies; AD/MCI+, Alzheimer’s dementia and mild cognitive impairment with evidence of amyloid pathology; ACE-R, Addenbrookes cognitive examination (revised) PK, [^11^C]-PK11195; SD, standard deviation.

Figure 5 displays the loadings of the three principal components as a heatmap and as colours on a template brain. [^11^C]-PK11195 (PK) component 1 primarily represented the cingulate, medial frontal, medial occipital (including the cuneus and lingula) and parietal areas (green); PK component 2 represented primarily hippocampal, parahippocampal, anterior temporal, frontal and lateral occipital regions (red), with PK component 3 representing the subcortical regions (blue). A full list and heatmap of component loadings can be found in Supplementary Fig. 5 and Supplementary Table 9.

To test for the association between patterns of [^11^C]-PK11195 binding and cognitive decline, we looked at the interaction between individual component scores and time of ACE-R in a linear mixed-effects model, controlling for age, sex and baseline cognition, with the individual as a random effect. Previous work in this cohort has shown that [^11^C]-PK-11195 binding in the anterior temporal lobes predicts cognitive decline in the AD/MCI+ group.^33^ We therefore tested for this association using the principal components above in order to compare this directly with the DLB group. This analysis replicated our previous finding; specifically, PK component 2 (representing hippocampal and anterior temporal lobes) was associated with cognitive decline in AD/MCI+ (beta −1.63, *P* = 0.01). Also consistent with our previous results, there were no significant associations with PK components 1 (beta = −1.30, *P* = 0.10) or 3 (beta = −0.11, *P* = 0.87) (Supplementary Tables 10 and 11). In contrast to AD/MCI+, in DLB participants, higher levels of PK component 1 (representing increased TSPO binding potential in the cingulate, cuneus, with some frontal and parietal regions, as shown in green in Fig. 5) were associated with a slower rate of cognitive decline (beta = 3.0, *P* = 0.033), whilst higher levels of PK component 2 (representing increased TSPO binding potential in the hippocampus, parahippocampus, orbitofrontal cortex and other regions, as shown in red in Fig. 5) were associated with a faster rate of cognitive decline (beta = −4.82, *P* = 0.03). There was no significant association with component 3, which represented basal ganglia and subcortical regions (beta = 0.34, *P* = 0.84).

### [11C]-PK11195 binding was associated with peripheral inflammatory markers in AD/MCI+

Finally, we wanted to identify the relationship between the regional [^11^C]-PK-11195 binding potential and the peripheral inflammatory markers and whether this differed between DLB and AD/MCI+. In this sample, there were no significant correlations between peripheral inflammatory markers and [^11^C]-PK11195 components after correction for multiple comparisons, either in all groups combined or separately. Bayesian correlations did not find strong support for either the null or alternative hypothesis that [^11^C]-PK11195 and peripheral inflammatory marker components were correlated (Supplementary Table 12). As an exploratory analysis, we used partial least squares regression (PLS-R), as this allows prediction of a set of *Y* variables (peripheral markers) from a set of *X* variables (regional binding potential of [^11^C]-PK11195) and identifies latent variables that best capture the covariance between predictors and responses. We included the 40 bilateral regions of [^11^C]-PK11195 binding as *X* predictor variables, and 30 inflammatory markers as *Y* response variables separately in the DLB and AD/MCI+ groups. In DLB, this identified a single component (Supplementary Fig. 6 and Tables 13 and 14) explaining 61.9% of the [^11^C]-PK11195 variance, but only 3.4% of the peripheral inflammatory marker variance, and a single significant region-cytokine pair (IL-27 and inferior-lateral anterior temporal lobe, estimate = 0.013, *P* = 0.044). This combined PLS component was not associated with cognitive decline in DLB (Supplementary Table 18). In AD/MCI+, one component was selected that explained 40.7% of the variance in [^11^C]-PK11195 binding, spanning the temporal, frontal and parietal lobes. The peripheral inflammatory marker variance depended on the cytokine, with an *R*^2^ of 0.32 for MCP4, 0.29 for TARC and 0.17 for Eotaxin-1 (Supplementary Fig. 7 and Tables 15 and 16). Significant pairs of [^11^C]-PK11195 regions and peripheral inflammatory markers are displayed in Supplementary Fig. 7 and Supplementary Table 17. This combined peripheral inflammation and [^11^C]-PK11195 component was a significant predictor of cognitive decline in AD/MCI+ (*t* = 4.176, *P* < 0.0001; Supplementary Fig. 8 and Supplementary Table 19).

## Discussion

In this study, we tested the hypothesis that baseline peripheral inflammatory markers and central neuroinflammation predict cognitive decline in DLB. Firstly, we identified individual peripheral cytokines, chemokines and other inflammatory markers associated with both a greater (IL-15, IL-6, YKL-40, Eotaxin-3, IL12p70) and slower (MDC, TNFR1, M-CSF1, IL16, GM-CSF, IL-12, MIP-3α, TNF-beta and IP10) rate of cognitive decline. We compared this with AD/MCI+, where no single marker withstood correction for multiple comparisons. Using PCA, we then identified a pattern of peripheral inflammatory markers that was associated with a slower rate of cognitive decline in DLB and AD/MCI+ (primarily loaded with TNFR1, IL-12, TNF-alpha and IP10), and a second pattern associated with a greater rate of cognitive decline in AD/MCI+ (primarily loaded with MCP4, Eotaxin-3, Eotaxin-1, and TARC). Next, we identified a pattern of [^11^C]-PK11195 binding associated with a slower rate of cognitive decline in DLB (cingulate, cuneus, with some frontal and parietal regions) and a pattern associated with a greater rate of cognitive decline in both DLB and AD/MCI+ (hippocampus and parahippocampus). Finally, using PLS-R, we identified in AD/MCI+ a shared profile including both the peripheral inflammatory markers and TSPO regions that together was a stronger predictor of more rapid cognitive decline than either the peripheral cytokines or TSPO separately. This contrasts with DLB, where there were very limited associations between TSPO binding and peripheral cytokines.

Consistently with our results, the peripheral inflammatory markers associated with more rapid decline in DLB included a range of pro-inflammatory cytokines. Firstly, IL-15 is of interest as a therapeutic target for autoimmune disorders given its role in CD8 T-cell homeostasis and NK cell activation.^61^ CD8 T-cell infiltration to the substantia nigra is an early event in synucleinopathy.^62^ NK cells are increased in the blood in Parkinson's disease, are associated with α-synuclein in post-mortem Lewy body disease (LBD) cases and act as scavengers for α-synuclein; depletion in animal models exacerbates synuclein pathology.^63^ IL-12 and IL-6 are involved in T-helper-1 (Th1) cell and T-helper-17 (Th17) cell activation and differentiation, respectively, with IL-6 inhibiting activation of T-regulatory cells^64^; these cell types are targets for immunomodulatory therapy in autoimmune diseases.^65^ There is a Th1 bias in peripheral T-cells in Parkinson's disease,^66^ whilst other studies report a shift to increased Th17 cells and decreased regulatory T-cells^67^ (T-reg). IL-17 producing T-cells are associated with Lewy bodies in LBD brains.^68^ YKL-40 (also known as chitinase-3-like-protein or CHI3L1) is expressed in astrocytes and association with microglial activation, but also elevated in the periphery in a range of inflammatory and malignant diseases, has been extensively studied in the context of Alzheimer’s disease.^69^ In contrast to Alzheimer's disease, YKL-40 is not increased in the CSF in DLB^70^ unless in the context of an Alzheimer's disease biomarker profile,^36^ but plasma levels have been shown to be increased compared to controls.^71^ CSF YKL-40 has been shown to predict longitudinal cognitive decline in Alzheimer's disease,^72^ but there are no studies previously linking YKL-40 to cognitive decline in DLB.

Conversely, we also found inflammatory markers associated with a slower progression of cognitive decline in DLB, some of which are associated with immune regulation. This is again broadly consistent with the data presented here. For example, sTNFR1 secretion attenuates systemic inflammation^73^ by acting as a decoy receptor for TNF-alpha.^74^ MDC^75,76^ and IL-16^77^ are associated with T-reg recruitment, and GM-CSF is being developed as a therapeutic in Parkinson's disease to increase T-reg cells.^78^ The contrast of IL-12p70 associated with more rapid cognitive decline and IL12 (p40) associated with a slower cognitive decline can be understood in the context of the bioactivity of IL-12 being dependent on the ratio between the active pro-inflammatory IL12p70 subunit and the antagonistic IL12p40 subunit.^79^

A longitudinal study including both MCI due to Lewy bodies (MCI-LB) and MCI-AD cases found the reduction of IFN-γ, IL1β, IL2, IL4, IL6 and IL10 over time to be associated with increased cognitive decline^30^ which could represent immune exhaustion during disease progression. This presents an alternative hypothesis to why higher levels of numerous cytokines are associated with slower rates of cognitive decline in this cohort, as this may be capturing an earlier disease stage, although in this study, higher ACE-R scores were not associated with higher cytokine levels.^25^

The relationship between TSPO PET and cognitive decline has not previously been studied in DLB. We identified regional patterns associated with both reduced and increased cognitive decline. In Alzheimer's disease, whilst several studies have identified increased TSPO PET in temporal regions as associated with a faster rate of cognitive decline,^33,34^ others have found a more complex relationship depending on the stage of disease.^80^ Microglia have a homeostatic and protective role^81^ but respond to the underlying neuropathology to distinct activation states^82^ which can be associated with inflammatory cytokine release. TSPO PET has been reflective of phagocytic microglia^83^ or microglial number rather than activation.^84^ It could be that TSPO PET across different regions is capturing a range of microglial states depending on the local pathology, and this is reflected in the differing associations with cognitive decline. Novel PET tracers that better capture the range of microglial states may help disentangle the underlying microglial profiles.^85^ This may also be that microglial activation is more pronounced in early stages of DLB.^25^ The medial temporal lobe was a key TSPO binding region associated with a greater rate of cognitive decline. Future studies should explore whether this or other regions identified in our study represent areas with the highest levels (or longest exposure) to Lewy body^86^ or other co-pathologies.^87^ It will also be important to understand how distinct microglial profiles in these regions^15,88^ may be associated with disease progression.

In AD/MCI+, no single peripheral inflammatory marker after correction for multiple comparisons was associated with cognitive decline. However, by using PCA, we identified a pattern in both DLB and AD/MCI+ (component 1) associated with a slower rate of cognitive decline, including many of the cytokines discussed above (such as TNFR1 and IL12). A separate profile (component 2) was associated with more rapid cognitive decline in AD/MCI+. This component represented MCP4, Eotaxin3, Eotaxin1, IL6 and MCP1. Eotaxin1 and Eotaxin3 bind to CCR3 and are classically associated with eosinophil chemoattraction. More recently, Eotaxins have been associated with ageing, Alzheimer's disease and reduced neurogenesis in mice.^89^ Small molecule antagonists of CCR3 are associated with improvements in Alzheimer's disease mouse models,^90^ and there is genetic evidence suggesting Eotaxins play a casual role in Alzheimer's disease.^91^ MCP4 and MCP1 are chemokines that bind to the CCR2 receptor; cerebrospinal fluid (CSF) levels of MCP1 are associated with Alzheimer's disease^92^ and progression from MCI to Alzheimer's dementia.^29^ MCP1 is produced in the brain by microglia, astrocytes and blood-derived macrophages^93^ and has been associated with increased amyloid^94^ and tau accumulation in animal models,^95^ although other studies have suggested this pathway is necessary for microglia accumulation and prevention of pathology.^96^ We performed an exploratory analysis to see if this component also predicted cognitive decline in DLB with high levels of amyloid co-pathology as measured by PiB PET. This showed a significant effect when using centiloid as a continuous variable (*P* = 0.012) but with only a trend as a binary cut-off (*P* = 0.068) Neuropathology studies have shown that neuroinflammation in DLB is dependent on the presence of amyloid beta and *p*-tau,^37^ and future studies are required to delineate whether the relationship between neuroinflammation and cognitive decline is dependent on co-pathology in DLB.

The association between TSPO binding in anterior temporal regions and cognitive decline in AD/MCI+ has previously been demonstrated in this cohort.^33^ Other studies have found differential effects of TSPO on disease progression depending on disease stage and region.^97^ Few studies have explored the associations between TSPO PET and peripheral inflammation,^98^ though recent studies in Parkinson's disease found IL6, IL1β-beta and IFN γ were associated with TSPO binding^35^ and proinflammatory cytokines associated with TSPO binding in frontal and brainstem regions in fronto-temporal lobar degeneration.^99^ Consistently, in our study, we found a relationship between regional TSPO binding and peripheral chemokines (i.e. MCP1, MCP4 and Eotaxin1) that was associated with cognitive decline. Likewise, there was a relationship between TSPO and TARC, which is a chemokine in animal models expressed by immune-challenged hippocampal neurons^100^ and in Alzheimer's disease animal models.^101^ Importantly, we identified that combining measures of central and peripheral inflammation led to the strongest associations with cognitive decline in AD/MCI+. This contrasted with DLB, where we found very limited associations between TSPO binding and peripheral inflammatory markers although as suggested by Bayesian correlations this study may have been underpowered to detect these associations.

This study had some limitations. This was a clinically defined cohort, and participants did not have confirmation of α-synuclein pathology. This is now becoming available with seed amplification assays which demonstrate high sensitivity and specificity to detect Lewy body pathology *in vivo*.^102^ The application of the clinical consensus DLB criteria has been validated in autopsy cohorts^103^ with high specificity, but future studies would benefit from direct measurement of α-synuclein. MCI participants underwent additional testing for the presence of Alzheimer’s pathology with amyloid PET imaging, whilst Alzheimer's dementia participants were recruited based on clinical criteria without the necessity for biomarkers. This has been validated in autopsy cohorts,^104^ but future studies would benefit from biomarker confirmation in all cases. Controls similarly were recruited based on the absence of cognitive symptoms, clinical assessment and neuropsychological testing rather than based on the absence of Alzheimer’s pathology, so we cannot exclude in this group Alzheimer’s pathology without clinical manifestations. Future studies would benefit from combined clinical and biomarker definitions of case and control participants.

The median length of follow-up was 2 years for DLB participants and 3 years for AD/MCI+ participants. This means there was variation in the number of follow-ups individuals underwent due to dropout. We used linear mixed-effects models as they are robust to dropouts when missingness is random^105^; however as dropouts were primarily due to individuals dying or being untestable, the results are likely limited to predicting decline in this cohort prior to that stage of disease. Future studies would benefit from more longitudinal data by recruiting participants at the MCI or early stages of disease, inclusion of cognitive tests that could be completed in more severe disease (such as the Montreal Cognitive Assessment) and longitudinal informant measures. Larger studies would also allow alternative models to incorporate the influence of dropout on the results.^106^

This study was a single site, and modest in sample size and the results must be interpreted in that context and taken provisionally. Future multi-site or combined cohort studies will be needed to both increase the statistical power and include diverse representative participants, to further test these associations. Of particular note is the influence of Alzheimer’s co-pathology on immune processes in DLB and larger studies stratified by the presence of amyloid co-pathology are required to robustly test for these effects.

Secondly, several peripheral inflammatory markers (11 out of 41, ∼27%) had greater than 50% measurements below the limit of detection and were excluded, which could mean important inflammatory pathways were not captured. We partially mitigated against these effects by using multiple imputation on inflammatory marker measurements with less than 50% missing values. Whilst the majority of included cytokines had low missingness, IL12p70 was close to the 50% threshold with 44.6% missing, and thus, this result should be treated with caution. The PCA identified three main components that explained 34% of the variance in the data, suggesting that there are likely additional relationships and complexities not captured by the PCA results. This is reflected in our analysis of individual cytokines which identified relationships with inflammatory markers (such as IL15) that are not well represented in any component. This, combined with samples being below the limit of detection, is an important limitation and further studies could use novel assays with increased sensitivity and a greater range of inflammatory markers to explore the full profile of the immune responses.^107,108^ Furthermore, the time of day and food ingestion were not standardized in these samples. Future studies would benefit from a standardized collection time to rule out these potential confounds.^109^

Next, whilst this study had longitudinal cognitive follow-up, peripheral cytokines and TSPO PET were only measured at baseline. As studies including DLB^25^ and prodromal stages^30^ have suggested an early peak in inflammation, the association between specific increased peripheral cytokines and slower cognitive decline may better capture clinical presentation at an early disease stage, rather than being representative of a protective immune response. Similarly, in AD/MCI+, the impact of TSPO signal on disease progression may vary with disease stage.^110,111^ Further, the DLB group did not include MCI cases, and the average ACE-R was lower than the AD/MCI+ group; thus, differences between the two diseases could also be explained by disease stage. It is likely that peripheral and neuroinflammation are dynamic processes through the disease, and future studies with multiple biomarker sampling points encompassing the spectrum from MCI to dementia are required. There is also further work required to better understand the relationship between peripheral and central inflammation: although we did not find significant associations between TSPO PET and peripheral inflammatory markers, we also did not find strong evidence for a lack of association, which likely reflects the power of the study. Neuroinflammation could also be anatomically inconsistent across the group, such as depending on disease stage.^25^ Due to the small number of female participants in the DLB group, we would also not be able to detect sex-specific effects,^112^ which may be relevant for neuroinflammation.^113^ Larger studies would also allow the inclusion of random slopes in predictive models to better capture individual variation in cognitive decline. Future studies should incorporate multiple time point measures of peripheral and central inflammation, alongside clinical assessments, and incorporate prodromal disease stages, such as MCI-LB,^114^ alongside measures of co-pathology.

TSPO PET has been extensively studied across neurodegenerative disorders,^115^ with strong evidence of specific regional patterns of inflammation,^33,116^ associations with pathology^13,117^ and outcomes.^35,99^ Studies have questioned whether TSPO better represents microglial number rather than activation^84^ and it is evident that TSPO PET cannot capture the full range of microglial states associated with neurodegenerative disorders.^15^ Future studies could use novel PET tracers to a greater range of inflammatory targets.^85^

Finally, we did not identify the source of the peripheral inflammatory profiles. Whilst we excluded individuals from recruitment with intercurrent infection, inflammatory or autoimmune diseases and further excluded from this analysis individuals with raised hsCRP, we cannot fully exclude subclinical infectious or other comorbidities associated with low-level inflammation as the source. We do identify specific pathways in DLB previously associated with immune cells in Parkinson's disease, rather than a role for general systemic inflammation, and in AD/MCI+ identify associations between peripheral and central inflammation.

This gives a clear direction for future studies: longitudinal cohorts including those at early or prodromal disease stages, with multiple time points of biomarker sampling. Studies need to incorporate measures of both peripheral and central inflammation, and novel technologies are expanding the range and accuracy of measurements to capture specific immune profiles, including proteomics and PET ligands. Beyond measuring soluble inflammatory markers, further studies are identifying specific cell populations dysregulated in DLB^118,119^ to better describe the role of the immune system in DLB and identify novel targets for therapeutics.

## Supplementary Material

fcag274_Supplementary_Data

## Data Availability

Anonymized processed data can be shared upon request to the corresponding or senior authors. Raw data may also be requested but are likely to be subject to a data transfer agreement with restrictions required to comply with participant consent and data protection regulations. Analyses were performed in *R* using standard functions and packages as described in the methods, and no new code or packages were generated.
