# Supplementary material for: Peripheral and central inflammation associated with progressive cognitive decline in dementia with Lewy bodies
Source: Brain Commun. 2026 Jul 14;8(4):fcag274. doi: 10.1093/braincomms/fcag274 (PMC13416190; doi:10.1093/braincomms/fcag274)

Supplementary table 1: details of assays for serum inflammatory markers

| **Inflammatory marker** | **Assay platform** |
| --- | --- |
| TNFR1 (CD120a), IL- 17A Gen. B,  IL-21, IL-22, IL-23, IL-27, IL-31, CCL20 (MIP-3α), GM-CSF, IL-1α, IL-12, IL-15,  IL-16, IL-17A, IL-5, IL-7, TNF-β, VEGF,  CCL11 (Eotaxin), CCL26 (Eotaxin 3), CXCL10 (IP10), CCL2 (MCP-1), CCL13 (MCP-4), CCL22 (MDC), CCL3 (MIP1α),  CCL4 (MIP1β), CCL17 (TARC), IFN-γ, IL-10, IL-12p70, IL-13, IL-1β, IL-2, IL-4, IL-6, IL-8, Tumour necrosis factor alpha (TNF-α) | Mesoscale Discovery V-Plex Human Cytokine 36 plex panel |
| High sensitivity CRP (hsCRP) | Siemens Dimension EXL autoanalyser |
| Interleukin-34 (IL-34)  YKL-40 (chitinase-3-like protein 1) | Bio-Techne R&D Systems kit |
| Macrophage Colony stimulating factor 1 (MCSF1) | Mesoscale discovery electrochemiluminescence immunoassay. |

Supplementary figure 1: Spaghetti plot showing the raw ACE-R scores over time in the DLB (n=20) and AD/MCI+ (n=30) groups. The y-axis sows the ACE-R score and the x-axis time from baseline in years. Each point represents a cognitive score at a time from baseline for an individual and are joined with separate coloured lines. (DLB=dementia with Lewy bodies, AD/MCI+=Alzheimer’s disease and mild cognitive impairment with evidence of amyloid positivity, ACE-R=Addenbrookes Cognitive Examination (Revised)).


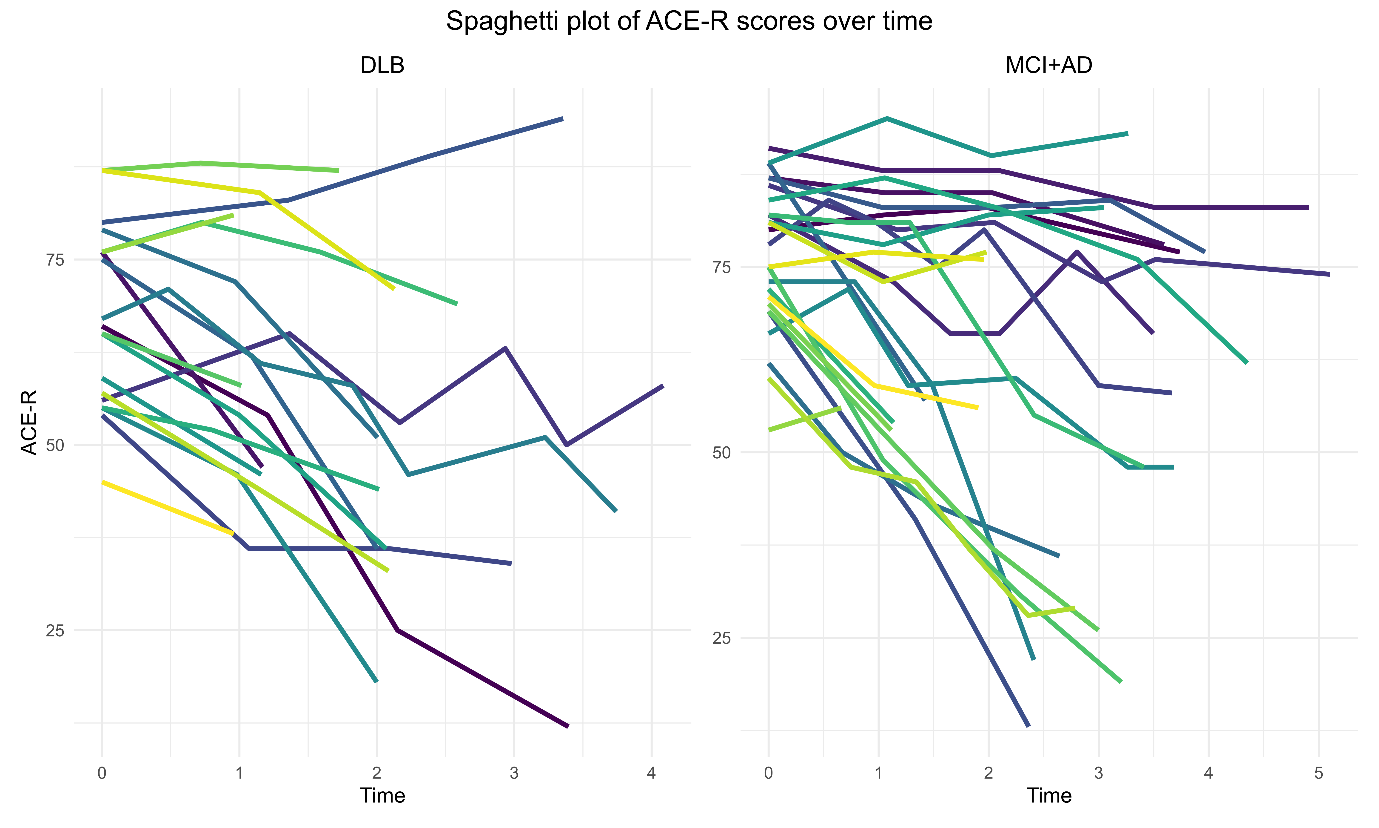


Supplementary table 2: Proportion of values below the lower limit of detection in cytokine measurements.

| Inflammatory marker | Percent below the LLOD |
| --- | --- |
| hsCRP | 6.8 |
| IL-34 | 100.0 |
| YKL40 | 1.4 |
| MCSF1 | 0.0 |
| TNFR1 | 0.0 |
| IL-17A-B | 93.2 |
| IL-21 | 97.3 |
| IL-22 | 18.9 |
| IL-23 | 100.0 |
| IL-27 | 0.0 |
| IL-31 | 95.9 |
| MIP-3-α | 5.4 |
| G-MCSF | 2.7 |
| IL-1α | 83.8 |
| IL-12 | 0.0 |
| IL-15 | 0.0 |
| IL-16 | 0.0 |
| IL-17A | 2.7 |
| IL-5 | 63.5 |
| IL-7 | 0.0 |
| TNF-β | 0.0 |
| VEGF | 0.0 |
| Eotaxin-1 | 0.0 |
| Eotaxin-3 | 4.1 |
| IP10 | 0.0 |
| MCP-1 | 0.0 |
| MCP-4 | 0.0 |
| MDC | 0.0 |
| MIP-1-α | 1.4 |
| MIP-1-β | 0.0 |
| TARC | 0.0 |
| IFN-gamma | 0.0 |
| IL-10 | 23.0 |
| IL-12p70 | 44.6 |
| IL-13 | 52.7 |
| IL-1β | 100.0 |
| IL-2 | 56.8 |
| IL-4 | 91.9 |
| IL-6 | 0.0 |
| IL-8 | 0.0 |
| TNF-α | 0.0 |

Supplementary figure 2: Box and scatter plots for log of cytokine concentration in picograms per ml between groups. Uncorrected p-values are shown for the Kruskal-Wallis test (DLB=dementia with Lewy bodies, MCI_AD=Alzheimer’s disease and mild cognitive impairment with evidence of amyloid positivity, GMCSF=granulocyte macrophage colony stimulation factor, IFNgamma = interferon gamma, IL=interleukin, IP10=inducible protein 10, MCP=monocyte chemoattractant protein, MCSF=macrophage colony stimulating factor, MDC= macrophage derived chemokine, MIP=macrophage inflammatory protein, TARC=thymus activation regulated chemokine, TNF=tumour necrosis factor, VEGF=vascular endothelial growth factor, YKL40=chitinase-like protein 3, pg/ml=picograms per mililitre).


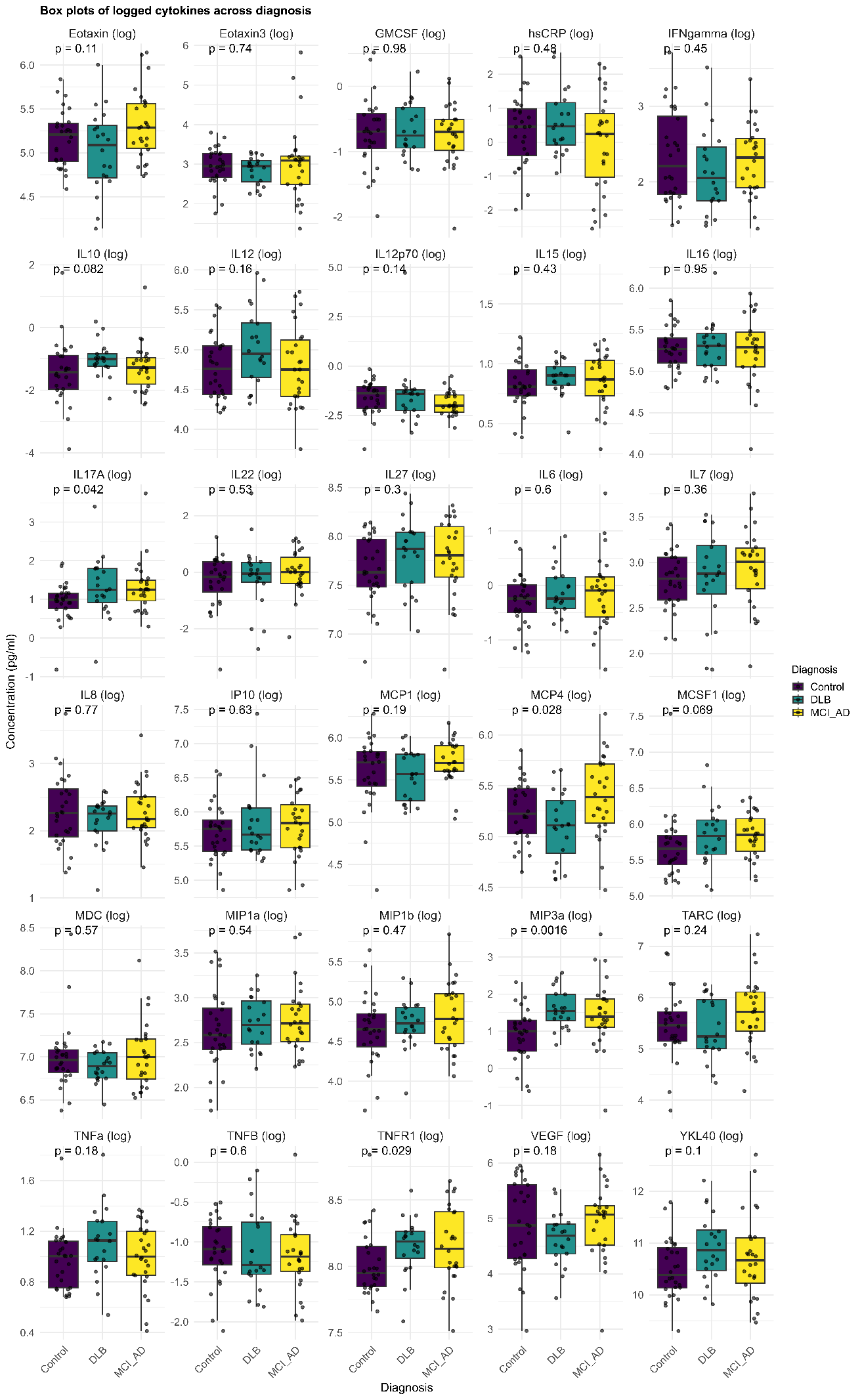


Supplementary table 3&4: Results for the interaction between cytokines and cognitive decline in DLB and AD/MCI+ from linear mixed effects models.

Supplementary table 3: Association between individual cytokines and cognitive decline in DLB from linear mixed effects models, p-value for the interaction of the individual cytokine and time.

| Parameter | Estimate | p-value | p-value (FDR) |
| --- | --- | --- | --- |
| YKL40 | -6.10 | <0.005 | <0.005 |
| MCSF1 | 15.26 | <0.005 | <0.005 |
| TNFR1 | 24.28 | <0.005 | <0.005 |
| IL27 | -2.68 | 0.46 | 0.52 |
| IL12 | 8.54 | <0.005 | <0.005 |
| IL15 | -33.71 | <0.005 | <0.005 |
| IL16 | 13.34 | <0.005 | <0.005 |
| IL7 | -3.17 | 0.10 | 0.16 |
| TNFB | 4.87 | 0.01 | 0.03 |
| VEGF | -2.54 | 0.32 | 0.40 |
| Eotaxin | -1.75 | 0.46 | 0.52 |
| IP10 | 4.43 | 0.02 | 0.04 |
| MCP1 | 6.22 | 0.15 | 0.22 |
| MCP4 | 1.62 | 0.66 | 0.68 |
| MDC | 30.79 | <0.005 | <0.005 |
| MIP1b | 5.41 | 0.22 | 0.29 |
| TARC | 1.25 | 0.51 | 0.54 |
| IFNgamma | 5.38 | 0.04 | 0.07 |
| IL6 | -6.60 | <0.005 | 0.01 |
| IL8 | -1.11 | 0.75 | 0.75 |
| TNFa | 6.63 | 0.08 | 0.13 |
| hsCRP | -1.92 | 0.14 | 0.22 |
| IL22 | -3.27 | 0.05 | 0.08 |
| MIP3a | 5.96 | <0.005 | <0.005 |
| GMCSF | 9.18 | <0.005 | 0.01 |
| IL17A | 1.59 | 0.19 | 0.26 |
| Eotaxin3 | -6.82 | 0.02 | 0.04 |
| MIP1a | 5.86 | 0.13 | 0.21 |
| IL10 | -1.39 | 0.47 | 0.52 |
| IL12p70 | -4.12 | <0.005 | 0.01 |

Supplementary table 4: Association between individual cytokines and cognitive decline in AD/MCI+ from linear mixed effects models, p-value for the interaction of the individual cytokine and time.

| Parameter | Estimate | p-value | p-value (FDR) |
| --- | --- | --- | --- |
| YKL40 | 1.11 | 0.15 | 0.25 |
| MCSF1 | 1.29 | 0.59 | 0.68 |
| TNFR1 | 6.88 | 0.02 | 0.09 |
| IL27 | 1.83 | 0.37 | 0.51 |
| IL12 | 1.43 | 0.42 | 0.55 |
| IL15 | 7.04 | 0.06 | 0.16 |
| IL16 | 4.84 | 0.00 | 0.06 |
| IL7 | 1.11 | 0.57 | 0.68 |
| TNFB | 0.30 | 0.88 | 0.88 |
| VEGF | -2.29 | 0.02 | 0.09 |
| Eotaxin | -5.09 | 0.01 | 0.08 |
| IP10 | 3.67 | 0.03 | 0.11 |
| MCP1 | -6.57 | 0.01 | 0.08 |
| MCP4 | -4.84 | 0.00 | 0.06 |
| MDC | 4.85 | 0.02 | 0.09 |
| MIP1b | -3.94 | 0.03 | 0.11 |
| TARC | -1.32 | 0.33 | 0.48 |
| IFNgamma | 2.85 | 0.08 | 0.18 |
| IL6 | 1.71 | 0.13 | 0.23 |
| IL8 | -3.44 | 0.10 | 0.19 |
| TNFa | 3.06 | 0.24 | 0.38 |
| hsCRP | 1.05 | 0.07 | 0.16 |
| IL22 | 0.95 | 0.34 | 0.48 |
| MIP3a | 0.18 | 0.86 | 0.88 |
| GMCSF | 0.65 | 0.66 | 0.72 |
| IL17A | 3.22 | 0.07 | 0.16 |
| Eotaxin3 | -1.69 | 0.04 | 0.13 |
| MIP1a | 1.42 | 0.51 | 0.64 |
| IL10 | -1.80 | 0.11 | 0.20 |
| IL12p70 | -0.59 | 0.67 | 0.72 |

Supplementary figure 3: Gene-concept network plot for significant cytokines associated with cognitive decline in DLB.

Using inflammatory markers in DLB (n=20) participants significantly associated with rate of cognitive decline from linear mixed effects models we tested for significantly enriched pathways in the KEGG database. Significance is tested with the hypergeometric test followed by FDR correction with only significant pathways (p<0.05) displayed in the diagram. Small circles represent the genes corresponding to the inflammatory marker measured, and larger circles represent disease or signalling pathways significantly enriched from the KEGG database. Lines connect the genes to the pathways. Red pathways indicate those associated with a faster rate of cognitive decline, and blue associated with a slower rate of cognitive decline. Pathways shared between rates of decline are ‘Cytokine-cytokine receptor interaction’, ‘Viral protein interaction with cytokine and cytokine receptor’, ‘JAK-STAT signalling pathway’ and ‘COVID-19’, whilst ‘IL17 signalling pathway’ was associated with a slower rate of decline, and ‘Malaria’, ‘African trypanosomiasis’ and ‘Intestinal immune network for IgA production’ associated with more rapid cognitive decline. (DLB= dementia with Lewy bodies, KEGG=Kyoto Encyclopaedia of Genes and Genomes, FDR= false discovery rate).


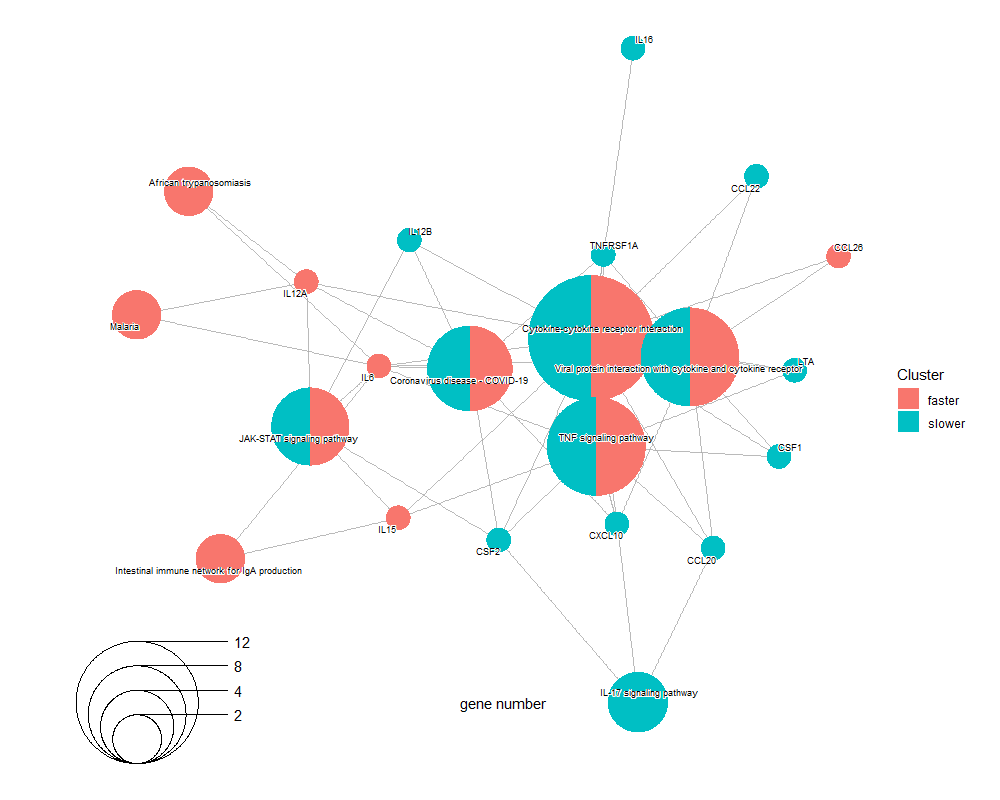


Supplementary figure 4: Gene-concept network plot for significant cytokines associated with cognitive decline in AD/MCI+.

As no inflammatory markers were significant after correcting for multiple comparisons in AD/MCI+ (n=26), raw p-values were used for selection of inflammatory from linear mixed effects models. These inflammatory markers were tested for significant enrichment in KEGG pathways. Significance is tested with the hypergeometric test followed by FDR correction with only significant pathways (p<0.05) displayed in the diagram. Small circles represent the genes corresponding to the cytokine measured, and larger circles represent disease or signalling pathways significantly enriched from the KEGG database. Lines connect the genes to the pathways. Red pathways are associated with cytokines significantly associated with a faster rate of cognitive decline, and those in blue associated with a slower rate of cognitive decline. Two associated pathways shared between rates of decline are ‘Cytokine-cytokine receptor interaction’, ‘Viral protein interaction with cytokine and cytokine receptor’, are shared with DLB, however ‘Chemokine signalling pathway is unique. ‘Influenza A’ and ‘Hepatitis C’ are associated with a slower rate of decline, and ‘Human Cytomegalovirus infection’ and ‘IL-17 signalling pathway’ are enriched in those cytokines associated with more rapid cognitive decline (AD/MCI+=Alzheimer’s disease and mild cognitive impairment, KEGG=Kyoto Encyclopaedia of Genes and Genomes, FDR=false discovery rate, DLB=dementia with Lewy bodies).


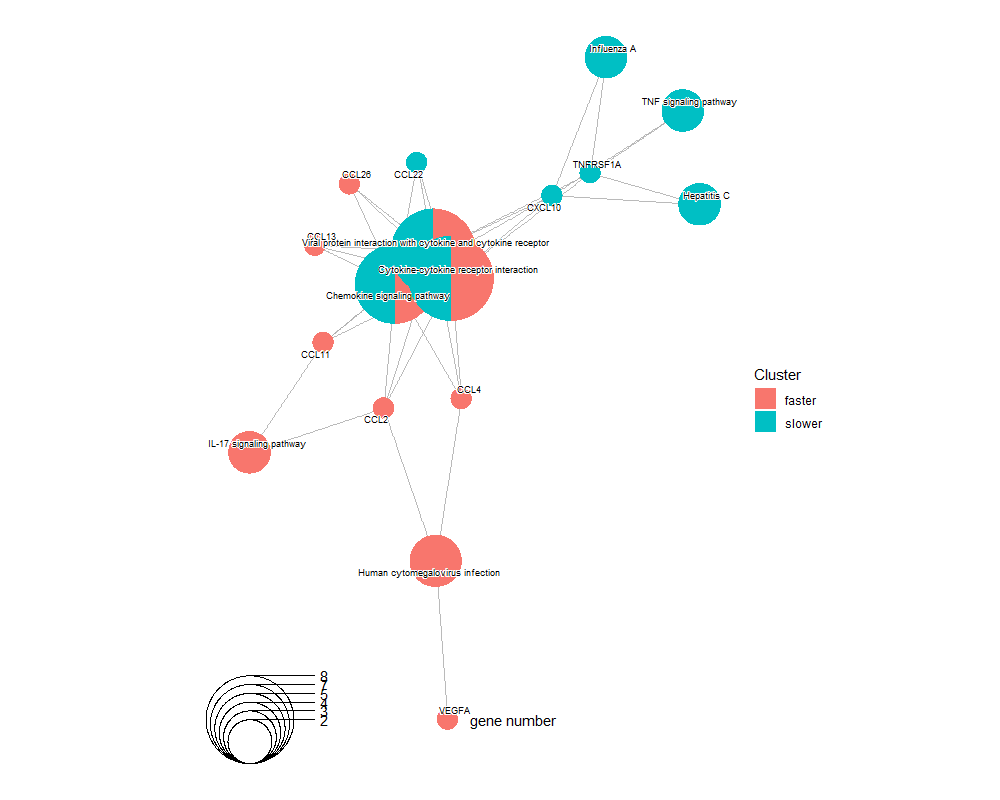


Supplementary table 5: Loadings (varimax rotated) for the principal component analysis of cytokines

| Cytokine | PC1 | PC2 | PC3 |
| --- | --- | --- | --- |
| TNFR1 | 0.74 | 0.23 | 0.12 |
| IL12 | 0.70 | 0.10 | -0.17 |
| TNFa | 0.67 | 0.23 | 0.26 |
| IP10 | 0.63 | -0.09 | -0.03 |
| IL27 | 0.57 | 0.26 | 0.09 |
| GMCSF | 0.56 | -0.12 | 0.03 |
| TNFB | 0.49 | -0.27 | 0.10 |
| MIP1a | 0.46 | 0.03 | 0.53 |
| IL22 | 0.44 | -0.01 | -0.25 |
| YKL40 | 0.43 | 0.09 | -0.44 |
| IL16 | 0.43 | -0.07 | 0.20 |
| MCSF1 | 0.41 | 0.23 | 0.45 |
| MIP1b | 0.40 | 0.05 | 0.61 |
| IL17A | 0.36 | 0.28 | -0.44 |
| IL6 | 0.33 | 0.53 | -0.05 |
| IFNgamma | 0.32 | -0.10 | 0.03 |
| MCP1 | -0.27 | 0.50 | 0.06 |
| hsCRP | 0.24 | 0.13 | 0.32 |
| TARC | -0.23 | 0.59 | 0.21 |
| MIP3a | 0.20 | 0.38 | 0.03 |
| IL10 | 0.19 | 0.03 | 0.07 |
| Eotaxin | -0.17 | 0.69 | 0.19 |
| IL12p70 | -0.11 | -0.14 | -0.12 |
| MDC | -0.08 | 0.30 | -0.11 |
| Eotaxin3 | 0.07 | 0.72 | -0.15 |
| IL15 | 0.06 | 0.13 | 0.04 |
| VEGF | -0.05 | 0.15 | 0.66 |
| MCP4 | 0.03 | 0.74 | 0.13 |
| IL8 | 0.02 | 0.20 | 0.48 |
| IL7 | 0.00 | -0.12 | 0.34 |

Supplementary table 6: Linear mixed effects model results in DLB with cytokine component scores as predictors of cognitive decline (PC1 = component scores from principal component 1, PC2 = principal component 2, PC3 = principal component 3), showing a significant interaction of PC1 with time on ACE-R scores. (SE = standard error, CI = confidence interval, df = degrees of freedom).

| Parameter | Coefficient | SE | CI_low | CI_high | Statistic | df_error | p |
| --- | --- | --- | --- | --- | --- | --- | --- |
| (Intercept) | 63.52 | 27.32 | 8.63 | 118.42 | 2.33 | 49.11 | 0.02 |
| time | -9.20 | 1.36 | -11.94 | -6.46 | -6.75 | 49.11 | <0.005 |
| PC1 | 0.00 | 2.39 | -4.80 | 4.80 | 0.00 | 49.11 | 1.00 |
| PC2 | 1.62 | 3.72 | -5.86 | 9.11 | 0.44 | 49.11 | 0.66 |
| PC3 | -1.31 | 3.23 | -7.81 | 5.19 | -0.40 | 49.10 | 0.69 |
| EntryAge | -0.80 | 0.40 | -1.61 | 0.01 | -2.00 | 49.11 | 0.05 |
| SexMale | -7.34 | 5.57 | -18.54 | 3.85 | -1.32 | 49.11 | 0.19 |
| ACER_baseline | 1.02 | 0.17 | 0.68 | 1.37 | 6.04 | 49.11 | <0.005 |
| time:PC1 | -3.02 | 0.94 | -4.90 | -1.14 | -3.23 | 48.97 | <0.005 |
| time:PC2 | -2.44 | 1.51 | -5.47 | 0.60 | -1.61 | 49.09 | 0.11 |
| time:PC3 | -2.23 | 1.80 | -5.84 | 1.38 | -1.24 | 49.04 | 0.22 |

Supplementary table 7: Linear mixed effects model results in AD/MCI+ with cytokine component scores as predictors of cognitive decline (PC1 = component scores from principal component 1, PC2 = principal component 2, PC3 = principal component 3), showing a significant interaction of PC1 with time, and PC2 with time on ACE-R scores. SE = standard error, CI = confidence interval, df = degrees of freedom).

| Parameter | Coefficient | SE | CI_low | CI_high | Statistic | df_error | p |
| --- | --- | --- | --- | --- | --- | --- | --- |
| (Intercept) | -31.97 | 33.67 | -98.86 | 34.93 | -0.95 | 89.03 | 0.34 |
| time | -5.53 | 0.69 | -6.90 | -4.15 | -7.99 | 89.05 | <0.005 |
| PC1 | 3.65 | 3.78 | -3.85 | 11.15 | 0.97 | 89.04 | 0.34 |
| PC2 | -0.61 | 2.00 | -4.59 | 3.36 | -0.31 | 89.05 | 0.76 |
| PC3 | 0.98 | 2.65 | -4.29 | 6.25 | 0.37 | 89.04 | 0.71 |
| EntryAge | 0.33 | 0.36 | -0.38 | 1.04 | 0.92 | 89.05 | 0.36 |
| SexMale | -3.37 | 5.46 | -14.22 | 7.48 | -0.62 | 89.05 | 0.54 |
| ACER_baseline | 1.12 | 0.26 | 0.60 | 1.65 | 4.27 | 89.04 | <0.005 |
| time:PC1 | -2.17 | 0.85 | -3.86 | -0.47 | -2.53 | 89.05 | 0.01 |
| time:PC2 | -1.78 | 0.79 | -3.35 | -0.22 | -2.27 | 89.04 | 0.03 |
| time:PC3 | 0.98 | 0.82 | -0.65 | 2.61 | 1.20 | 88.99 | 0.23 |

Supplementary table 8: Linear mixed effects model results in DLB with cytokine component scores as predictors of cognitive decline, including the interaction with PiB status (PC1 = component scores from principal component 1, PC2 = principal component 2, PC3 = principal component 3), showing a significant interaction of PC2 with PiB status and time on ACE-R scores. (SE = standard error, CI = confidence interval, df = degrees of freedom).

| Parameter | Coefficient | SE | statistic | df | p.value |
| --- | --- | --- | --- | --- | --- |
| (Intercept) | 59.65 | 27.80 | 2.15 | 8.35 | 0.06 |
| time | -1.75 | 3.48 | -0.50 | 35.09 | 0.62 |
| PC1 | 1.99 | 2.75 | 0.72 | 14.86 | 0.48 |
| PC2 | -5.39 | 6.93 | -0.78 | 13.34 | 0.45 |
| PiB_status_CLPositive | 13.17 | 8.22 | 1.60 | 15.35 | 0.13 |
| PC3 | 1.08 | 3.29 | 0.33 | 17.63 | 0.75 |
| EntryAge | -0.88 | 0.43 | -2.06 | 7.98 | 0.07 |
| SexMale | -9.77 | 5.68 | -1.72 | 8.71 | 0.12 |
| ACER_baseline | 1.01 | 0.16 | 6.23 | 8.39 | 0.00 |
| time:PC1 | 1.65 | 1.17 | 1.41 | 35.71 | 0.17 |
| time:PC2 | 3.16 | 2.85 | 1.11 | 34.59 | 0.28 |
| time:PiB_status_Positive | -8.39 | 3.68 | -2.28 | 35.65 | 0.03 |
| PC2:PiB_status_Positive | 9.09 | 7.48 | 1.22 | 14.06 | 0.24 |
| time:PC3 | 3.95 | 1.87 | 2.12 | 36.50 | 0.04 |
| time:PC2:PiB_status_Positive | -13.06 | 4.02 | -3.25 | 38.12 | <0.01 |

Supplementary figure 5: Heatmap showing the loadings of the three components of regional [11-C]-PK11195 binding from a principal components analysis performed in all participants with PET imaging (n=66). Each column represents a principal component (PC1 = principal component 1, PC2 = principal component 2, PC3 = principal component 3) and each row weighted average bilateral regions from the Hammer’s atlas. The colour of the cell represents the loading of each inflammatory marker on that component, with red indicating strong positive loading, and blue indicating strong negative loading.


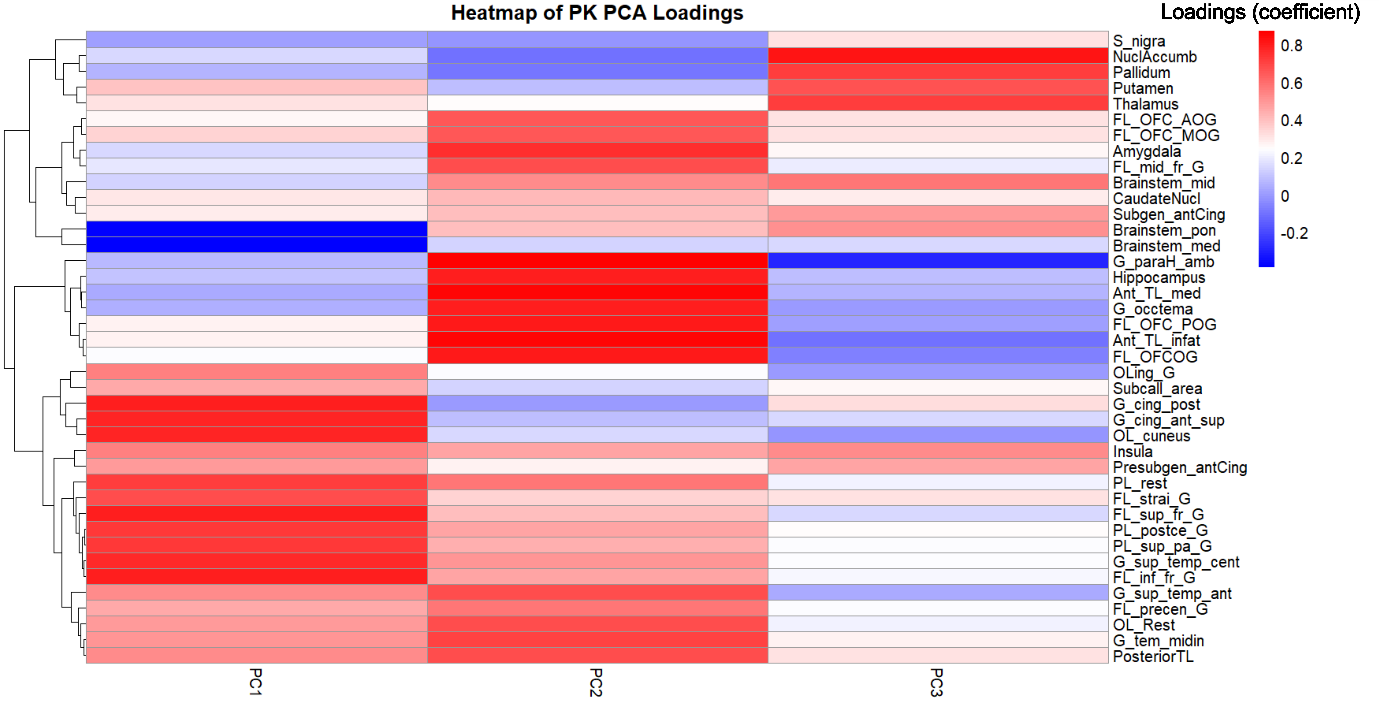


Supplementary table 9: Loadings from the principal component analysis of [11-C]-PK11195 regional binding potentials

| Region | PC1 | PC2 | PC3 |
| --- | --- | --- | --- |
| Hippocampus | 0.10 | 0.79 | 0.08 |
| Amygdala | 0.15 | 0.75 | 0.26 |
| Ant_TL_med | 0.03 | 0.85 | 0.06 |
| Ant_TL_infat | 0.27 | 0.85 | -0.11 |
| G_paraH_amb | 0.07 | 0.87 | -0.29 |
| G_sup_temp_cent | 0.76 | 0.50 | 0.23 |
| G_tem_midin | 0.49 | 0.70 | 0.28 |
| G_occtem | 0.05 | 0.79 | 0.00 |
| Insula | 0.55 | 0.46 | 0.52 |
| OL_rest | 0.48 | 0.68 | 0.21 |
| G_cing_ant_sup | 0.78 | 0.09 | 0.15 |
| G_cing_post | 0.79 | -0.01 | 0.32 |
| FL_mid_fr_G | 0.18 | 0.67 | 0.19 |
| PosteriorTL | 0.53 | 0.68 | 0.31 |
| PL_rest | 0.71 | 0.57 | 0.21 |
| CaudateNucl | 0.29 | 0.41 | 0.28 |
| NuclAccumb | 0.14 | -0.11 | 0.81 |
| Putamen | 0.38 | 0.09 | 0.67 |
| Thalamus | 0.31 | 0.24 | 0.72 |
| Pallidum | 0.06 | -0.09 | 0.71 |
| FL_precen_G | 0.45 | 0.57 | 0.24 |
| FL_strai_G | 0.68 | 0.35 | 0.30 |
| FL_OFC_AOG | 0.26 | 0.65 | 0.30 |
| FL_inf_fr_G | 0.78 | 0.45 | 0.22 |
| FL_sup_fr_G | 0.79 | 0.39 | 0.15 |
| PL_postce_G | 0.72 | 0.46 | 0.24 |
| PL_sup_pa_G | 0.72 | 0.43 | 0.24 |
| OLing_G | 0.54 | 0.24 | 0.00 |
| OL_cuneus | 0.77 | 0.15 | -0.02 |
| FL_OFC_MOG | 0.34 | 0.65 | 0.30 |
| FL_OFCOG | 0.24 | 0.80 | -0.07 |
| FL_OFC_POG | 0.27 | 0.80 | 0.01 |
| S_nigra | 0.01 | -0.01 | 0.31 |
| Subgen_antCing | 0.28 | 0.39 | 0.48 |
| Subcall_area | 0.45 | 0.13 | 0.25 |
| Presubgen_antCing | 0.48 | 0.27 | 0.46 |
| G_sup_temp_ant | 0.52 | 0.67 | 0.04 |
| Brainstem_mid | 0.13 | 0.52 | 0.58 |
| Brainstem_pon | -0.38 | 0.39 | 0.51 |
| Brainstem_med | -0.39 | 0.13 | 0.14 |

Supplementary table 10: Linear mixed effects model results of the interaction between [^11^C]-PK11195 component scores and time on ACE-R in DLB, showing a significant interaction of PK component 1 (PK_PC1) and 2 (PK_PC2), and time. (PK = [^11^C]-PK11195, PC = principal component, df = degrees of freedom).

| Variable | Estimate | Std. Error | df | t value | Pr(>\|t\|) |
| --- | --- | --- | --- | --- | --- |
| (Intercept) | 54.42 | 24.78 | 15.48 | 2.20 | 0.04 |
| time | -8.34 | 1.45 | 45.34 | -5.77 | 0.00 |
| PK_PC1 | -3.38 | 2.39 | 29.30 | -1.42 | 0.17 |
| PK_PC2 | 4.71 | 3.87 | 30.61 | 1.22 | 0.23 |
| PK_PC3 | -0.64 | 3.56 | 26.51 | -0.18 | 0.86 |
| EntryAge | -0.73 | 0.34 | 16.35 | -2.14 | 0.05 |
| SexMale | -11.26 | 5.25 | 16.93 | -2.14 | 0.05 |
| ACER_baseline | 1.14 | 0.17 | 16.63 | 6.79 | <0.005 |
| time:PK_PC1 | 3.01 | 1.37 | 43.59 | 2.20 | 0.03 |
| time:PK_PC2 | -4.82 | 2.15 | 44.86 | -2.24 | 0.03 |
| time:PK_PC3 | 0.34 | 1.72 | 45.70 | 0.20 | 0.85 |

Supplementary table 11: Linear mixed effects model results of the interaction between [^11^C]-PK11195 component scores and time on ACE-R in AD/MCI+, showing a significant interaction of PK component 2 (PK_PC2) and time. (PK = [^11^C]-PK11195, PC = principal component, df = degrees of freedom).

| Variable | Estimate | Std. Error | df | t value | Pr(>\|t\|) |
| --- | --- | --- | --- | --- | --- |
| (Intercept) | -36.28 | 16.37 | 21.75 | -2.22 | 0.04 |
| time | -5.13 | 0.64 | 86.70 | -7.95 | <0.005 |
| PK_PC1 | -1.48 | 1.89 | 40.26 | -0.78 | 0.44 |
| PK_PC2 | 0.51 | 1.46 | 41.48 | 0.35 | 0.73 |
| PK_PC3 | 0.78 | 1.44 | 41.49 | 0.54 | 0.59 |
| EntryAge | 0.09 | 0.18 | 20.55 | 0.53 | 0.60 |
| SexMale | -0.04 | 2.92 | 19.38 | -0.01 | 0.99 |
| ACER_baseline | 1.38 | 0.16 | 21.11 | 8.46 | <0.005 |
| time:PK_PC1 | -1.30 | 0.79 | 93.53 | -1.65 | 0.10 |
| time:PK_PC2 | -1.63 | 0.66 | 89.10 | -2.49 | 0.01 |
| time:PK_PC3 | -0.11 | 0.66 | 90.23 | -0.17 | 0.87 |

Supplementary table 12: Spearman correlation coefficients between cytokine and PK11195 components (PC = principal component, PK = [11C]-PK11195, BF = Bayes Factor). Bayesian correlations were performed in R with correlationsBF from the ‘BayesFactor’ package. Bayes Factor between 1 and 3 provides ‘mild’ evidence, between 3 and 10 ‘moderate’, between 10 and 30 ‘strong’ and above 30 ‘very strong’ evidence for the alternative hypothesis (BF_10_) over the null hypothesis, or the null hypothesis (BF_01_) over the alternative. Bayesian analysis were performed with Pearson correlations, due to the challenges of estimating BF for Spearman correlations.

Spearman Correlations by Group between cytokine and PK components

| **Diagnosis** | **Cytokine component** | **PK component** | **correlation** | **p value** | **BF_10_** | **BF_01_** |
| --- | --- | --- | --- | --- | --- | --- |
| All | PC1 | PK_PC1 | -0.04 | 0.7459 | 0.42 | 2.41 |
| All | PC1 | PK_PC2 | -0.15 | 0.2405 | 0.49 | 2.03 |
| All | PC1 | PK_PC3 | -0.15 | 0.2547 | 1.02 | 0.98 |
| All | PC2 | PK_PC1 | -0.18 | 0.1690 | 0.45 | 2.20 |
| **All** | **PC2** | **PK_PC2** | **0.23** | **0.0698** | 1.07 | 0.93 |
| All | PC2 | PK_PC3 | -0.08 | 0.5522 | 0.30 | 3.38 |
| All | PC3 | PK_PC1 | -0.05 | 0.7099 | 0.58 | 1.73 |
| **All** | **PC3** | **PK_PC2** | **0.29** | **0.0220** | 1.96 | 0.51 |
| All | PC3 | PK_PC3 | -0.07 | 0.5988 | 0.33 | 3.06 |
| Control | PC1 | PK_PC1 | 0.03 | 0.9258 | 0.53 | 1.89 |
| Control | PC1 | PK_PC2 | -0.08 | 0.7629 | 0.52 | 1.92 |
| Control | PC1 | PK_PC3 | -0.14 | 0.5938 | 0.61 | 1.65 |
| Control | PC2 | PK_PC1 | -0.41 | 0.1144 | 1.06 | 0.94 |
| Control | PC2 | PK_PC2 | 0.33 | 0.2084 | 1.01 | 0.99 |
| Control | PC2 | PK_PC3 | -0.11 | 0.6725 | 0.52 | 1.91 |
| Control | PC3 | PK_PC1 | 0.12 | 0.6564 | 0.61 | 1.65 |
| Control | PC3 | PK_PC2 | -0.07 | 0.7882 | 0.55 | 1.83 |
| Control | PC3 | PK_PC3 | 0.24 | 0.3729 | 0.57 | 1.77 |
| DLB | PC1 | PK_PC1 | 0.26 | 0.2584 | 1.38 | 0.72 |
| DLB | PC1 | PK_PC2 | -0.02 | 0.9316 | 0.49 | 2.05 |
| DLB | PC1 | PK_PC3 | -0.03 | 0.8862 | 0.49 | 2.05 |
| DLB | PC2 | PK_PC1 | -0.19 | 0.4108 | 0.92 | 1.08 |
| DLB | PC2 | PK_PC2 | -0.15 | 0.5338 | 0.62 | 1.61 |
| DLB | PC2 | PK_PC3 | 0.09 | 0.7096 | 0.66 | 1.52 |
| DLB | PC3 | PK_PC1 | -0.23 | 0.3309 | 0.55 | 1.81 |
| DLB | PC3 | PK_PC2 | 0.15 | 0.5212 | 0.53 | 1.89 |
| DLB | PC3 | PK_PC3 | -0.09 | 0.7001 | 0.79 | 1.26 |
| MCI_AD | PC1 | PK_PC1 | -0.32 | 0.1172 | 0.61 | 1.63 |
| MCI_AD | PC1 | PK_PC2 | -0.23 | 0.2724 | 0.82 | 1.22 |
| MCI_AD | PC1 | PK_PC3 | -0.31 | 0.1296 | 2.70 | 0.37 |
| MCI_AD | PC2 | PK_PC1 | 0.01 | 0.9545 | 2.98 | 0.34 |
| MCI_AD | PC2 | PK_PC2 | 0.21 | 0.3213 | 0.56 | 1.78 |
| MCI_AD | PC2 | PK_PC3 | -0.00 | 0.9839 | 0.44 | 2.25 |
| MCI_AD | PC3 | PK_PC1 | -0.03 | 0.8961 | 0.92 | 1.09 |
| MCI_AD | PC3 | PK_PC2 | 0.23 | 0.2578 | 0.63 | 1.60 |
| MCI_AD | PC3 | PK_PC3 | 0.06 | 0.7726 | 0.44 | 2.28 |

Supplementary figure 6: Loadings for the PLS-R component in DLB (n=20). Panel A shows the [^11^C]-PK11195 loadings as colours on the MNI152 brain template. Panel B shows a heatmap of the peripheral inflammatory marker loadings, with each row representing a single inflammatory marker. The colour of the cell represents the loading of each inflammatory marker on that component, with red indicating strong positive loading, and blue indicating strong negative loading (PLS-R=partial least squares regression, DLB=dementia with Lewy bodies).


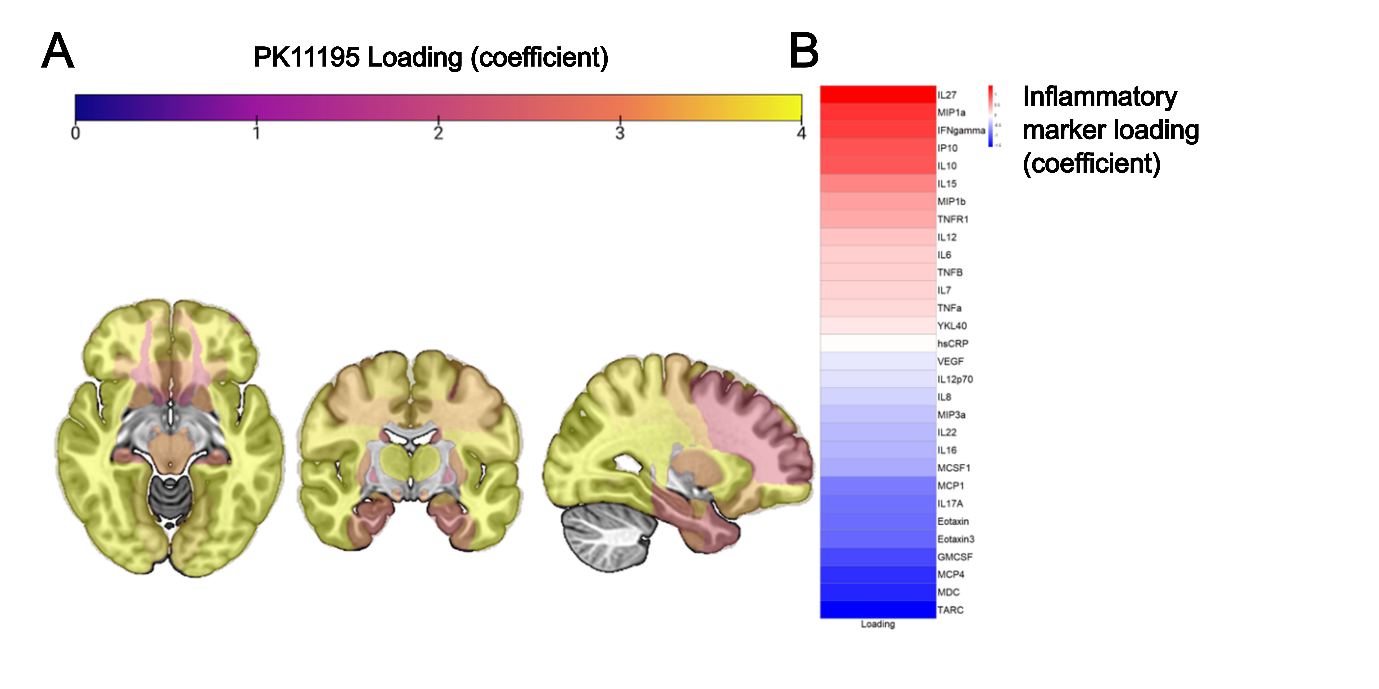


Supplementary table 13: Loadings for the first (and only) PLS-R component X values in DLB, representing the [^11^C]-PK11195 regional binding that best explained the variance in peripheral cytokines. Loadings were multiplied by -1 for interpretability.

| Variable | Comp 1 |
| --- | --- |
| PosteriorTL | 4.30 |
| G_sup_temp_cent | 4.20 |
| G_tem_midin | 4.18 |
| PL_rest | 4.16 |
| OL_rest | 4.15 |
| FL_inf_fr_G | 4.08 |
| OL_cuneus | 4.08 |
| FL_OFCOG | 4.07 |
| Ant_TL_infat | 4.02 |
| Insula | 4.02 |
| Thalamus | 4.02 |
| G_sup_temp_ant | 4.02 |
| FL_OFC_AOG | 3.89 |
| PL_postce_G | 3.86 |
| FL_strai_G | 3.81 |
| PL_sup_pa_G | 3.79 |
| FL_sup_fr_G | 3.76 |
| OLing_G | 3.76 |
| Presubgen_antCing | 3.63 |
| G_cing_post | 3.56 |
| Amygdala | 3.53 |
| FL_OFC_POG | 3.44 |
| FL_precen_G | 3.43 |
| FL_OFC_MOG | 3.34 |
| Brainstem_mid | 3.29 |
| Putamen | 3.25 |
| Subgen_antCing | 3.20 |
| G_occtema | 3.18 |
| Hippocampus | 2.96 |
| Subcall_area | 2.92 |
| G_cing_ant_sup | 2.72 |
| CaudateNucl | 2.68 |
| G_paraH_amb | 2.65 |
| Brainstem_pon | 2.53 |
| Ant_TL_med | 2.51 |
| FL_mid_fr_G | 2.40 |
| Pallidum | 2.14 |
| NuclAccumb | 1.94 |
| S_nigra | -0.19 |
| Brainstem_med | -0.38 |

Supplementary table 14: Loadings for the first (and only) PLS-R component Y values in DLB, representing the peripheral cytokine profile that was best explained the variance in [^11^C]-PK11195 regional binding. Loadings were multiplied by -1 for interpretability.

| Variable | Comp 1 |
| --- | --- |
| IL27 | 1.43 |
| MIP1a | 1.12 |
| IFNgamma | 1.05 |
| IP10 | 0.94 |
| IL10 | 0.89 |
| IL15 | 0.62 |
| MIP1b | 0.46 |
| TNFR1 | 0.42 |
| IL12 | 0.25 |
| IL6 | 0.20 |
| TNFB | 0.20 |
| IL7 | 0.17 |
| TNFa | 0.16 |
| YKL40 | 0.05 |
| hsCRP | -0.08 |
| VEGF | -0.20 |
| IL12p70 | -0.26 |
| IL8 | -0.33 |
| MIP3a | -0.43 |
| IL22 | -0.48 |
| IL16 | -0.52 |
| MCSF1 | -0.58 |
| MCP1 | -0.86 |
| IL17A | -0.92 |
| Eotaxin | -0.93 |
| Eotaxin3 | -0.97 |
| GMCSF | -1.15 |
| MCP4 | -1.31 |
| MDC | -1.36 |
| TARC | -1.59 |

Supplementary table 15: Loadings for the first (and only) PLS-R component X values in AD/MCI+, representing the [^11^C]-PK11195 regional binding that best explained the variance in peripheral cytokines. Loadings were multiplied by -1 for interpretability.

| Variable | Comp 1 |
| --- | --- |
| G_tem_midin | 4.43 |
| PL_rest | 4.15 |
| PL_postce_G | 4.09 |
| FL_inf_fr_G | 4.05 |
| G_sup_temp_cent | 4.03 |
| FL_sup_fr_G | 3.96 |
| G_sup_temp_ant | 3.89 |
| FL_precen_G | 3.84 |
| Insula | 3.75 |
| PosteriorTL | 3.74 |
| FL_OFC_MOG | 3.66 |
| PL_sup_pa_G | 3.62 |
| Ant_TL_infat | 3.61 |
| FL_OFCOG | 3.61 |
| FL_mid_fr_G | 3.60 |
| Ant_TL_med | 3.48 |
| FL_OFC_POG | 3.48 |
| OL_rest | 3.47 |
| FL_OFC_AOG | 3.30 |
| Amygdala | 3.23 |
| G_paraH_amb | 3.18 |
| Hippocampus | 3.09 |
| G_occtema | 3.03 |
| Putamen | 3.02 |
| FL_strai_G | 2.85 |
| CaudateNucl | 2.79 |
| Subgen_antCing | 2.69 |
| Brainstem_mid | 2.47 |
| G_cing_ant_sup | 2.45 |
| Thalamus | 2.30 |
| Presubgen_antCing | 2.22 |
| G_cing_post | 1.22 |
| OL_cuneus | 1.17 |
| Subcall_area | 0.95 |
| S_nigra | 0.77 |
| Pallidum | 0.76 |
| NuclAccumb | 0.67 |
| OLing_G | 0.49 |
| Brainstem_pon | -0.57 |
| Brainstem_med | -1.64 |

Supplementary table 16: Loadings for the first (and only) PLS-R component Y values in AD/MCI+, representing the peripheral cytokine profile that was best explained the variance in [^11^C]-PK11195 regional binding. Loadings were multiplied by -1 for interpretability.

| Variable | Comp 1 |
| --- | --- |
| MCP4 | 2.73 |
| TARC | 2.59 |
| MCP1 | 2.43 |
| Eotaxin | 1.95 |
| IL6 | 0.91 |
| hsCRP | 0.91 |
| MDC | 0.49 |
| MCSF1 | 0.38 |
| IL10 | 0.38 |
| TNFa | 0.36 |
| Eotaxin3 | 0.27 |
| MIP3a | 0.21 |
| IL8 | 0.15 |
| IL15 | 0.09 |
| VEGF | -0.16 |
| TNFB | -0.19 |
| IL12 | -0.19 |
| TNFR1 | -0.24 |
| IL27 | -0.45 |
| IL12p70 | -0.46 |
| IL7 | -0.59 |
| IL16 | -0.68 |
| MIP1a | -0.76 |
| IL22 | -0.81 |
| GMCSF | -0.83 |
| YKL40 | -0.91 |
| MIP1b | -0.94 |
| IL17A | -1.07 |
| IP10 | -1.21 |
| IFNgamma | -1.88 |


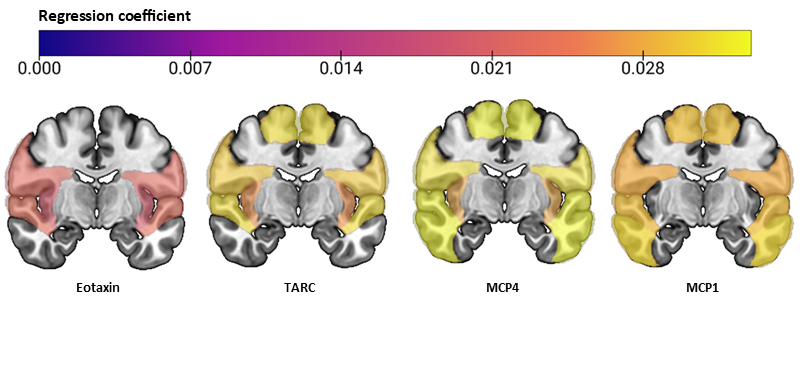
Supplementary figure 7: Regression coefficients for pairs of peripheral inflammatory markers and regions of [^11^C]-PK11195 binding from PLS-R in AD/MCI+ (n=25). The strength of the coefficient is displayed as the colour intensity of the region. Jackknife based 95% confidence intervals were calculated for each coefficient, and coefficients that did not include zero were considered statistically significant. Each brain plot shows the regression coefficient between [^11^C]-PK11195 region, plotted onto the MNI152 brain template, and one of four peripheral inflammatory markers with significant results. Only [^11^C]-PK11195 regions with a jackknife associated p-values less than 0.05, and 95% confidence intervals not including the null result, are displayed. The scalebar is unitless are the data was scaled but represents BPND per pg/mL (TARC=Thymus and Activation-Regulated Chemokine, MCP=monocyte chemoattractant protein, BPND=binding potential).

Supplementary table 17: Regression coefficients for the combination of peripheral cytokines and [^11^C]-PK11195 regional binding potentials from PLS-R in AD/MCI+, with only significant results displayed (based on jackknife confidence intervals).

| PK region | Cytokine | Estimated | Std. err. | t-value | p-value | 2.5% | 97.5% |
| --- | --- | --- | --- | --- | --- | --- | --- |
| PK_PLest | MCP4 | 0.031 | 0.009 | 3.543 | 0.002 | 0.013 | 0.050 |
| PK_PL_postce_G | MCP4 | 0.031 | 0.010 | 3.028 | 0.006 | 0.010 | 0.052 |
| PK_PLest | MCP1 | 0.028 | 0.010 | 2.849 | 0.009 | 0.008 | 0.048 |
| PK_PL_sup_pa_G | MCP4 | 0.029 | 0.010 | 2.843 | 0.009 | 0.008 | 0.050 |
| PK_G_sup_temp_cent | MCP4 | 0.033 | 0.011 | 2.817 | 0.010 | 0.009 | 0.056 |
| PK_G_tem_midin | MCP4 | 0.035 | 0.012 | 2.735 | 0.012 | 0.009 | 0.060 |
| PK_PL_postce_G | TARC | 0.030 | 0.011 | 2.625 | 0.015 | 0.007 | 0.053 |
| PK_PL_sup_pa_G | TARC | 0.027 | 0.010 | 2.618 | 0.015 | 0.006 | 0.049 |
| PK_G_sup_temp_cent | TARC | 0.031 | 0.012 | 2.585 | 0.017 | 0.006 | 0.056 |
| PK_Insula | TARC | 0.026 | 0.010 | 2.553 | 0.018 | 0.005 | 0.048 |
| PK_G_tem_midin | MCP1 | 0.031 | 0.012 | 2.470 | 0.021 | 0.006 | 0.056 |
| PK_PL_postce_G | MCP1 | 0.028 | 0.011 | 2.444 | 0.023 | 0.005 | 0.051 |
| PK_PL_sup_pa_G | Eotaxin | 0.021 | 0.009 | 2.375 | 0.026 | 0.003 | 0.038 |
| PK_PL_postce_G | Eotaxin | 0.022 | 0.009 | 2.375 | 0.026 | 0.003 | 0.042 |
| PK_Insula | MCP4 | 0.028 | 0.012 | 2.370 | 0.027 | 0.004 | 0.052 |
| PK_PLest | TARC | 0.030 | 0.013 | 2.321 | 0.029 | 0.004 | 0.056 |
| PK_FL_sup_fr_G | TARC | 0.031 | 0.013 | 2.311 | 0.030 | 0.004 | 0.059 |
| PK_PL_sup_pa_G | MCP1 | 0.026 | 0.011 | 2.303 | 0.031 | 0.003 | 0.048 |
| PK_FL_sup_fr_G | MCP4 | 0.033 | 0.014 | 2.282 | 0.032 | 0.003 | 0.063 |
| PK_G_sup_temp_cent | MCP1 | 0.029 | 0.013 | 2.279 | 0.032 | 0.003 | 0.055 |
| PK_Insula | Eotaxin | 0.020 | 0.009 | 2.220 | 0.037 | 0.002 | 0.038 |
| PK_PLest | Eotaxin | 0.022 | 0.010 | 2.151 | 0.042 | 0.001 | 0.044 |
| PK_FL_sup_fr_G | MCP1 | 0.029 | 0.013 | 2.149 | 0.042 | 0.001 | 0.057 |
| PK_G_sup_temp_cent | Eotaxin | 0.023 | 0.011 | 2.102 | 0.047 | 0.001 | 0.046 |

Supplementary table 18: Linear mixed effects model results of the interaction between the PLS-R component scores (scaled_sum) and time on ACE-R in DLB, showing no significant interaction with the component, representing a combination of [^11^C]-PK11195 regional binding and peripheral cytokines, and time. (df = degrees of freedom).

| Variable | Estimate | Std. Error | df | t value | Pr(>\|t\|) |
| --- | --- | --- | --- | --- | --- |
| (Intercept) | 60.67 | 21.88 | 15.92 | 2.77 | 0.01 |
| time | -5.67 | 1.13 | 54.95 | -5.03 | 0.00 |
| scaled_sum | -0.25 | 0.35 | 31.52 | -0.72 | 0.48 |
| EntryAge | -0.84 | 0.30 | 15.71 | -2.79 | 0.01 |
| SexMale | -12.20 | 4.44 | 17.01 | -2.75 | 0.01 |
| ACER_baseline | 1.14 | 0.15 | 18.78 | 7.36 | 0.00 |
| time:scaled_sum | 0.27 | 0.17 | 52.92 | 1.57 | 0.12 |

Supplementary table 19: Linear mixed effects model results of the interaction between the PLS-R component scores (scaled_sum) and time on ACE-R in AD/MCI+, showing a significant interaction the component, representing a combination of [^11^C]-PK11195 regional binding and peripheral cytokines, and time. (df = degrees of freedom).

| Variable | Estimate | Std. Error | df | t value | Pr(>\|t\|) |
| --- | --- | --- | --- | --- | --- |
| (Intercept) | -23.306 | 17.391 | 19.662 | -1.340 | 0.195 |
| time | -5.895 | 0.657 | 77.260 | -8.968 | 0.000 |
| scaled_sum | 0.001 | 0.184 | 30.698 | 0.003 | 0.997 |
| EntryAge | 0.069 | 0.187 | 17.293 | 0.370 | 0.716 |
| SexMale | -0.410 | 3.198 | 17.155 | -0.128 | 0.899 |
| ACER_baseline | 1.240 | 0.178 | 18.569 | 6.977 | <0.001 |
| time:scaled_sum | 0.275 | 0.066 | 79.155 | 4.162 | <0.001 |

Supplementary figure 8: Loadings for the PLS-R component in AD/MCI+ and association with longitudinal cognitive decline (n=25). Panel A shows the PK11195 loadings as colours on the MNI152 brain template. Panel B shows a heatmap of the peripheral cytokine loadings, with each row representing a single cytokine. The colour of the cell represents the loading of each cytokine on that component, with red indicating strong positive loading, and blue indicating strong negative loading. Coefficients are unitless as the data was scaled. Panel C shows interaction plots of the interaction between time and individual scores of the scaled sum of PLS-R scores from a linear mixed effect model (n=25), representing the combined pattern of PK11195 binding and peripheral cytokines as predictors of ACE-R scores in AD/MCI+. The y-axis shows ACE-R scores, and the x-axis shows time in years. The differences in the slope of the lines represents the difference in rate of cognitive decline at varying levels of PLS-R component scores (scaled_sum), controlling for age, sex, and baseline cognition. The lines shown represent the trajectories of the mean score, +1 and -1 standard deviation, with 95% confidence intervals. The effect size, beta, and p-value are shown for the interaction term. (PLS-R: partial least squares regression, AD/MCI+=Alzheimer’s disease and mild cognitive impairment, ACE-R=Addenbrookes Cognitive Examination Revised).


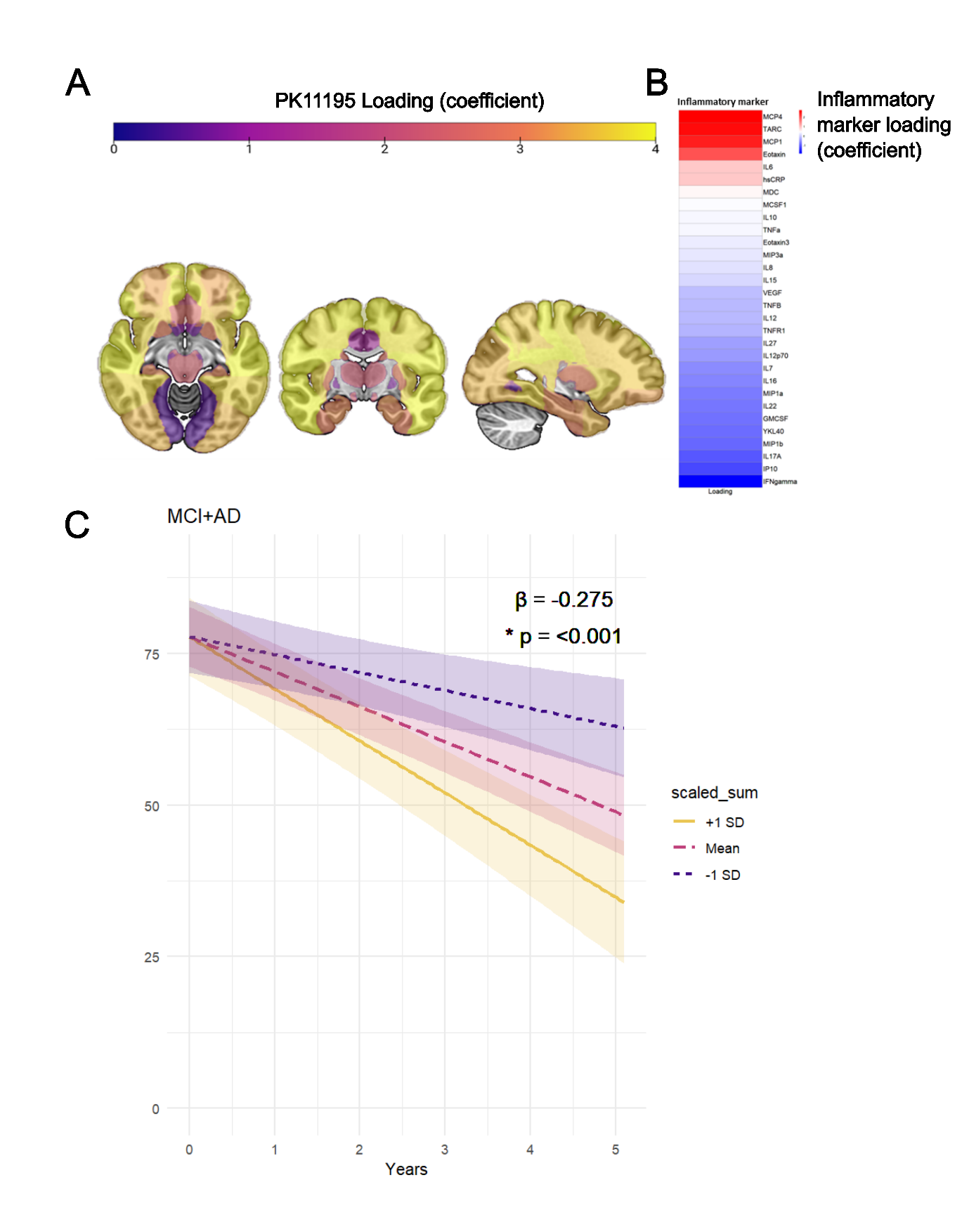

Supplement: fcag274_Supplementary_Data [file fcag274_supplementary_data.docx]
